# Including Photoexcitation Explicitly in Trajectory-Based Nonadiabatic Dynamics at No Cost

**DOI:** 10.1021/acs.jpclett.4c02549

**Published:** 2024-10-15

**Authors:** Jiří Janoš, Petr Slavíček, Basile F. E. Curchod

**Affiliations:** † Department of Physical Chemistry, University of Chemistry and Technology, Technická 5, Prague 6, 166 28, Czech Republic; ‡ Centre for Computational Chemistry, School of Chemistry, University of Bristol, Bristol BS8 1TS, United Kingdom

## Abstract

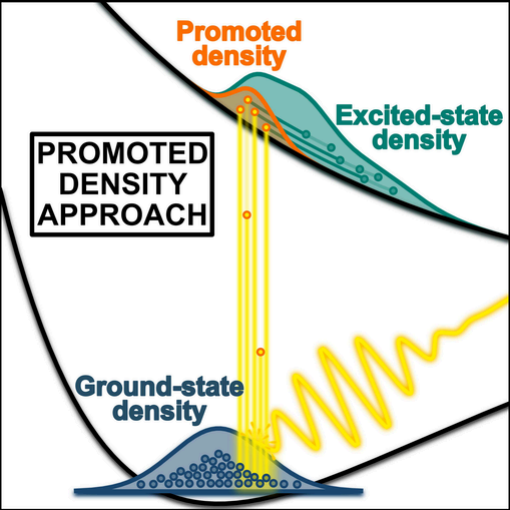

Over the last decades, theoretical photochemistry has produced multiple techniques to simulate the nonadiabatic dynamics of molecules. Surprisingly, much less effort has been devoted to adequately describing the first step of a photochemical or photophysical process: photoexcitation. Here, we propose a formalism to include the effect of a laser pulse in trajectory-based nonadiabatic dynamics at the level of the initial conditions, with no additional cost. The promoted density approach (PDA) decouples the excitation from the nonadiabatic dynamics by defining a new set of initial conditions, which include an excitation time. PDA with surface hopping leads to nonadiabatic dynamics simulations in excellent agreement with quantum dynamics using an explicit laser pulse and highlights the strong impact of a laser pulse on the resulting photodynamics and the limits of the (sudden) vertical excitation. Combining PDA with trajectory-based nonadiabatic methods is possible for any arbitrary-sized molecules using a code provided in this work.

Since the development of femtochemistry, the field of ultrafast spectroscopy has been flourishing, offering over the years a plethora of strategies to investigate the dynamics of photoexcited molecules.^1−4^ Current cutting-edge experiments performed at advanced light sources can probe photochemical reactions with precisely controlled femto/attosecond laser pulses and measure fine details of nuclear and sometimes even electronic evolution.^5^ This fast experimental pace has, of course, also greatly stimulated the development of theoretical approaches to describe the excited-state dynamics of molecules beyond the Born–Oppenheimer approximation, also called nonadiabatic molecular dynamics.^6−10^

Over the last decades, the field of nonadiabatic molecular dynamics has provided a large toolbox of methods for describing the evolution of molecules following photoexcitation to an excited electronic state,^11−13^ often based on trajectories when the molecule is treated in its full dimensionality.^10,14,15^ When combined with high-level electronic-structure techniques to describe the underlying electronic energies and nonadiabatic couplings, nonadiabatic molecular dynamics methods can be used to accurately model the excited-state dynamics of molecules and provide interpretation and guidance to experiments.

Yet, to describe a photochemical experiment reliably, it is crucial to account for all its steps: excitation of the molecule by a laser pulse (or light), evolution in the (coupled) excited electronic states, and formation of photoproducts (in the excited or ground electronic state). In contrast to the extensive developments in electronic-structure theory and nonadiabatic dynamics, it is striking to realize how primitive is the description of the photoexcitation process in current nonadiabatic simulations.^16^ As an example, let us consider a recent prediction challenge presented to the nonadiabatic-dynamics community, aiming at simulating the photochemistry of cyclobutanone and predicting the time-resolved ultrafast electron diffraction signal resulting from this dynamics before the experiment is conducted.^17^ While the experimentalists provided all the details about the laser pulse they would use for the photoexcitation of cyclobutanone, not a single group incorporated the pulse explicitly, including the authors of the present work. This observation can be rationalized by the associated computational cost of including a laser pulse explicitly in nonadiabatic dynamics simulations^18−20^ and the fact that including an explicit laser pulse tends to stretch the approximations of mixed quantum/classical methods like fewest-switches surface hopping (FSSH).^21,22^ However, it is still striking that almost half of the proposed predictions completely ignored the underlying effect of the laser pulse on the photoexcitation, that is, the energy spectrum and temporal spread of the pulse. The remaining predictions used a simple energy window based on the pulse spectrum to select initial conditions for the dynamics, yet with multiple definitions for the window. The pulse duration was also often ignored assuming instantaneous excitation. This recent observation, based on state-of-the-art simulations, reveals the following: *the field of nonadiabatic molecular dynamics critically lacks a standard approach to account for the photoexcitation by a laser pulse properly*.

In this Letter, we propose to establish a systematic scheme for the explicit inclusion of photoexcitation in nonadiabatic molecular dynamics simulations at the level of the initial conditions, with no additional cost. This strategy is derived from the concept of promoted nuclear density—and as such coined promoted density approach (PDA)—and can be straightforwardly applied to trajectory-based nonadiabatic molecular dynamics methods as it simply selects from the traditional initial conditions (nuclear positions and momenta) based on the laser pulse spectral intensity and complements them with an ’excitation time’. The PDA can also be used to justify a proper way of performing energy windowing and convolution in nonadiabatic dynamics.

PDA finds its origin in the derivation of a time-dependent excited-state nuclear density formula based on first-order perturbation theory, considering a weak field regime for the laser pulse. The first work on this topic was proposed by Martens et al.,^23^ further extended by Shen and Cina^24^ and later by Meyer and Engel,^25^ all considering a Gaussian laser pulse and constant transition dipole moment. Following their work, Martınez-Mesa and Saalfrank generalized the equation for an arbitrary pulse envelope using the pulse envelope Wigner representation.^26^ PDA builds on all the aforementioned works and encompasses any arbitrary laser pulse, as well as position-dependent transition dipole moments.

Let us discuss the key steps in the derivation that lead to PDA (a detailed derivation is proposed in the SI). We consider a molecular system with a ground (*g*) and an excited (*e*) electronic state, coupled through a weak interaction defined in the position representation as
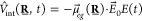
1where μ⃗_*eg*_ denotes the transition dipole moment depending on nuclear configuration **R**, *E⃗*_0_ is the electric field amplitude *E*_0_ multiplied by the polarization vector λ⃗ of the field, and *E*(*t*) is a real time-dependent electric field. The excited-state nuclear density for such a system can be expressed using first-order perturbation theory as
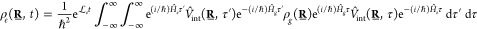
2where  is the excited-state quantum Liouville propagator defined as  and *Ĥ*_*g*/*e*_ = *T̂* + *E*_*g*/*e*_^el^(**R**) is the time-independent Hamiltonian for either the ground or excited electronic state with *E*_*g*/*e*_^el^(**R**) standing for the respective electronic potential energy surfaces (and *T̂* for the nuclear kinetic energy operator). We note that also the Liouvillian and Hamiltonian are position-dependent, but we drop **R** here to simplify the notation. The ground-state nuclear density, ρ_*g*_, is considered to be stationary under the *Ĥ*_*g*_ Hamiltonian.

The integrals in eq 2 can be disentangled by substituting τ = *t*′ + *s*/2 and τ′ = *t*′ – *s*/2, leading to
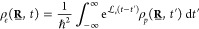
3where ρ_*p*_ is a promoted nuclear density, defined as
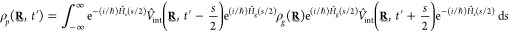
4Identifying the promoted density above allows us to separate the photoexcitation process (eq 4) from the excited-state dynamics (eq 3). The promoted nuclear density is the central quantity of our derivation and represents the nuclear density promoted to the excited electronic state at time *t*′, which we call the excitation time. As such, ρ_*p*_ is proportional to the interaction strength represented as *E*_0_ (see eq 1). The promoted nuclear density, once propagated in the excited electronic state from time *t*′ to *t* and integrated over all excitation times *t*′, reconstructs the correct excited-state density at time *t*, ρ_*e*_(**R**, *t*).

The expression for the promoted nuclear density, eq 4, can be simplified by applying twice the first-order Baker–Campbell–Hausdorff formula to the operators on both sides of the ground-state density ρ_*g*_ (see the SI for the detailed derivation and analysis of terms neglected), leading to
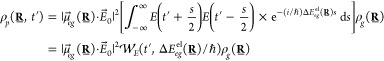
5where we have identified the term between squared brackets as the Wigner pulse representation ,^27^ defined as
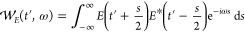
6The Wigner pulse representation can be viewed as a quasiprobability function for the laser pulse to have a certain pulse frequency at a given time, similar to the concept of a Wigner distribution in quantum mechanics. We will come back to the meaning and properties of  as soon as we finish this derivation.

Finally, inserting the promoted density into the expression for the excited-state nuclear density (eq 3) and taking a classical limit by retaining only the lowest-order term in ℏ in the Wigner transform of the density operator results in the following equation for PDA,
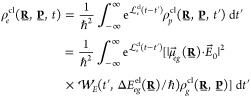
7where **P** stands for the nuclear momenta and  is the classical Liouvillian. This semiclassical approximation limits the formula to cases where the interference effects in the excited state can be ignored (see the SI).

Equation 7 as such is derived to describe the excited-state nuclear density only after the interaction with the laser pulse (see the SI for a detailed discussion). However, we can extend the reach of eq 7 such that it describes the excited-state density also during the pulse by altering the upper integration limit to *t* instead of ∞,
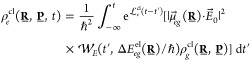
8This modification means that only the contributions of the promoted density to ρ_*e*_ coming from times before the current time *t* are included. Any contributions to ρ_*e*_ coming from the promoted density at later times *t*′ > *t* are neglected. This empirical alteration of the upper integration limit affects the overall equation only during the pulse interaction and not for times *t* after the pulse. This modification allows PDA to describe the excited-state nuclear density *within* the pulse duration while not altering the final excited-state density after the pulse. More details on this procedure (and its numerical validation) are provided in the SI.

Equation 8 provides a clear interpretation of the excitation process: for every time *t*′ of the laser pulse, the initial ground-state nuclear density is first multiplied by the transition dipole moment and the Wigner representation of the pulse and then promoted to the excited electronic state to be propagated from time *t*′ to a desired time *t*. The propagation is governed by the time-independent Hamiltonian for the excited electronic state, without the explicit laser pulse. Integration over all times *t*′ during the pulse until the current time *t* reconstructs the full excited-state nuclear density at time *t*, while the ground-state density remains unperturbed. This whole process is further illustrated in Figure 1.

**Figure 1:**
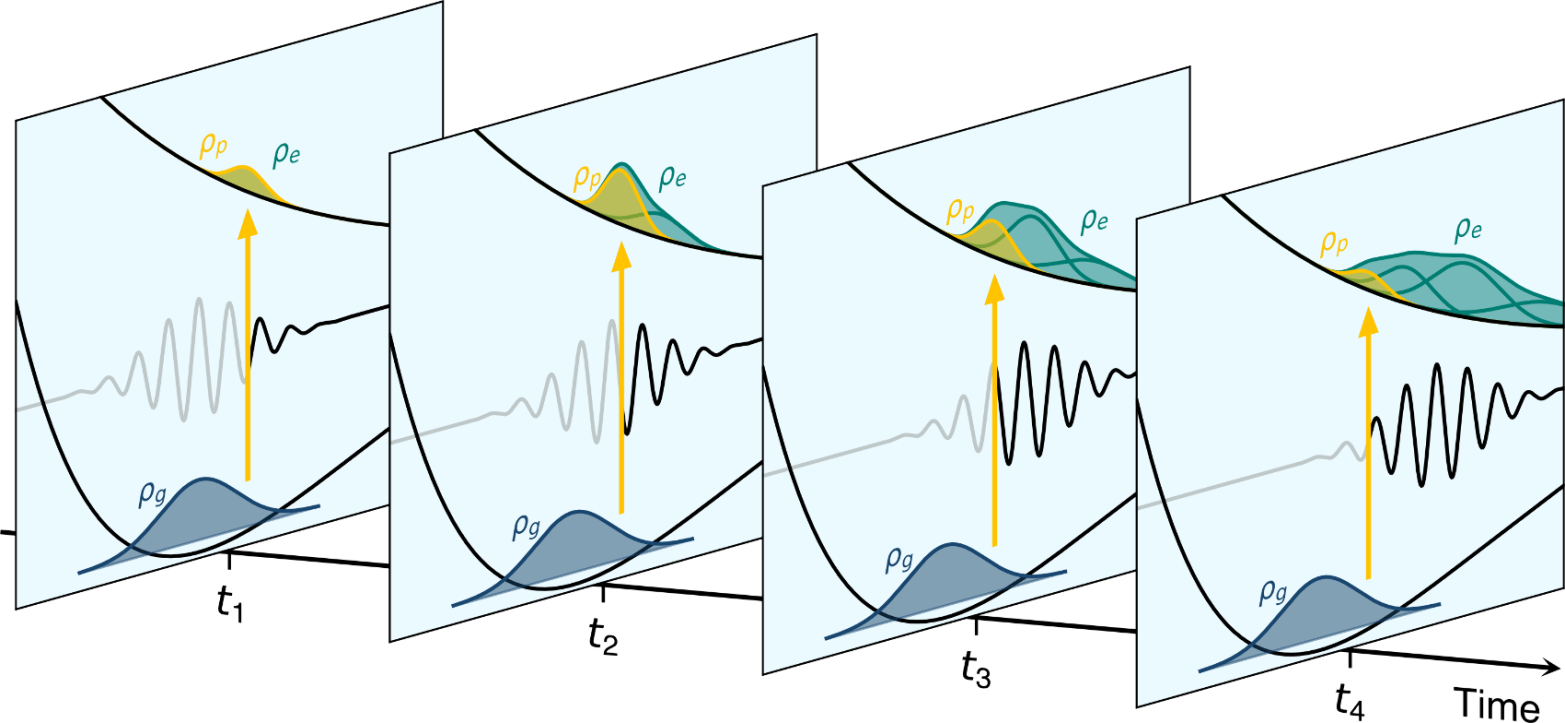
Illustration of photoexcitation and subsequent dynamics of the excited-state nuclear density ρ_*e*_ (green) depicted in terms of ground-state nuclear density ρ_*g*_ (blue) and promoted nuclear density ρ_*p*_ (yellow). Snapshots are given at four different times during the pulse interaction with a molecular system.

Hence, eq 8 offers a strategy to decouple the excitation process from the subsequent excited-state dynamics. By considering that the early times of the excited-state dynamics behave adiabatically, we can combine the description of photoexcitation discussed above to existing methods for excited-state molecular dynamics like fewest-switches surface hopping or *ab initio* multiple spawning. The only required additional step consists of sampling the promoted nuclear density ρ_*p*_^cl^(**R**, **P**, *t*′), but this is a simple task once we have the ground-state nuclear density, as we should soon see. At the end of our derivation, we would also like to state that the range of validity of eq 8 is limited and a detailed discussion about its assumptions and approximations is provided in the SI.

Before turning to the practical implementation of PDA, we briefly return as promised to the Wigner pulse representation (eq 6)—the key component of eq 8. Omitting their polarization, laser pulses can be either represented in the time domain as a time-dependent electric field *E*(*t*) or in the frequency domain as a pulse spectrum *Ẽ*(ω). These two representations are connected via a Fourier transform *Ẽ*(ω) = ∫_–∞_^∞^ *E*(*t*)e^–*iωt*^ d*t*. The Wigner pulse representation  provides a way to obtain simultaneous temporal and frequency information by representing the pulse in both the time and frequency domain. As its cousin from quantum mechanics, the Wigner pulse representation does not have a proper physical meaning as such, but its integrated forms provide important insights. In particular, integration over frequency
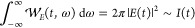
9yields the pulse intensity profile *I*(*t*)^a^ while integration over time
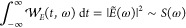
10gives the spectral intensity *S*(ω).^27^ Both *I*(*t*) and *S*(ω) are the quantities actually accessible experimentally (unlike *E*(*t*) and *Ẽ*(ω)), providing a more direct connection to laser experiments. Similarly to the Wigner representation of density,  can also acquire negative values making it rather quasiprobability than probability function. The occurrence of negative values hinders practical sampling from the distribution, yet we will discuss later that  is positive for the usual Gaussian envelope and only minor problems appear for other standard pulse envelopes.

Let us now illustrate some of the quantities discussed above with the practical example of sodium iodide (NaI) interacting with a Gaussian laser pulse. The parameters for the sodium iodide Hamiltonian are provided in the SI. We define the electric field of the Gaussian laser pulse as^b^
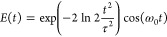
11where ω_0_ is the frequency of oscillations and τ corresponds to the full width at half-maximum (fwhm) parameter of the pulse intensity . We advocate for using the fwhm parameter for the intensity profile rather than for the field envelope ε (i) because the intensity is the experimentally accessible and reported quantity and (ii) because the population transfer to the excited electronic state is proportional to the intensity. Such a definition of τ hence minimizes disparities between experiments and theoretical investigations.

The Wigner pulse representation  for a specific pulse with τ = 20 fs and ℏω_0_ = 3.68 eV (corresponding to *ΔE*_*eg*_^el^ at a NaI distance of 2.74 Å) is represented in Figure 2A alongside the pulse intensity *I*(*t*) (panel B), spectral intensity *S*(ω) (panel C), and oscillating electric field *E*(*t*) (inset). The plotted  depicts the probability for a molecular geometry with excitation energy *ΔE* to be promoted to the excited state throughout the pulse duration. The spectral intensity for a 20 fs pulse is already quite narrow, targeting only a part of the NaI absorption spectrum. Thus, only a fraction of the ground-state probability density can potentially be excited.

**Figure 2:**
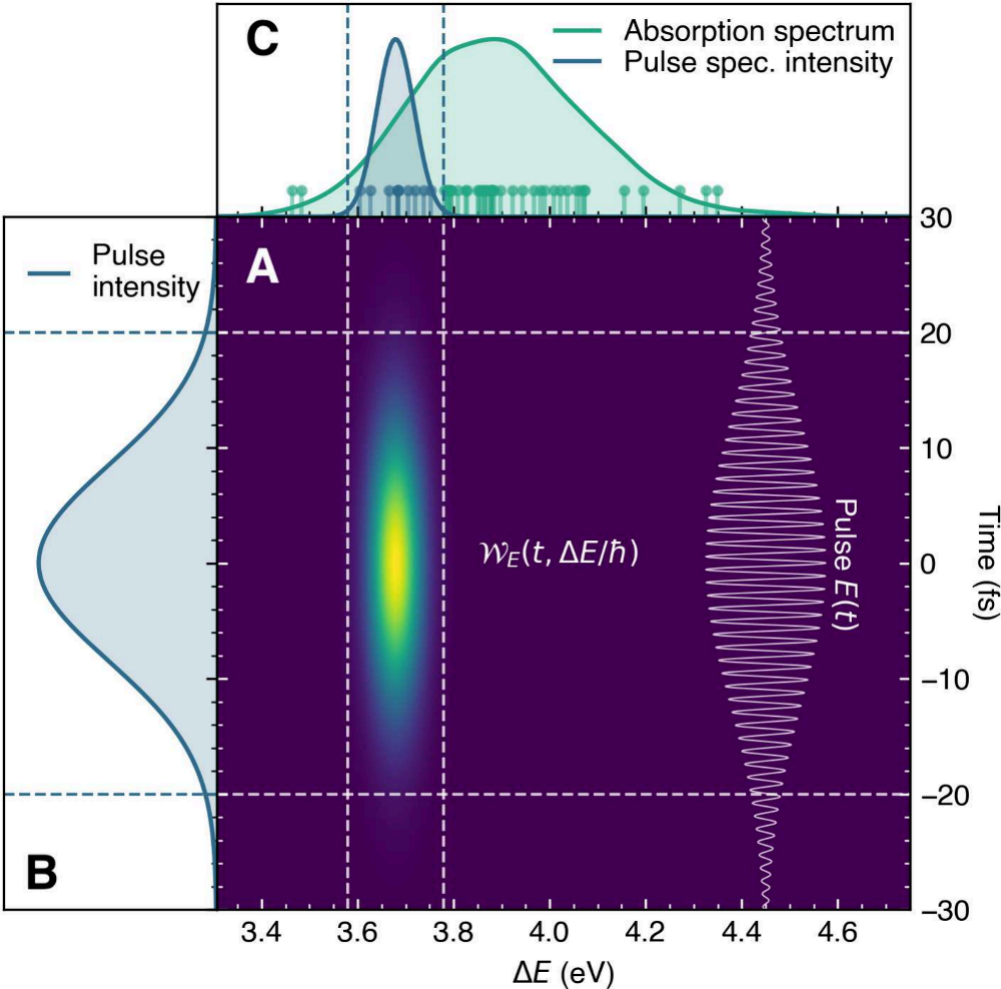
Illustration of the Wigner pulse representation and associated quantities for a laser pulse applied to sodium iodide. (A) Wigner pulse representation  for a Gaussian pulse with τ = 20 fs and ℏω_0_ = 3.6792 eV. Dashed lines correspond to a region containing twice the fwhm parameters of pulse intensity and spectral intensity. The pulse electric field is plotted as an inset. (B) Projection of  on the time axis (pulse intensity *I*). (C) Projection of  on the frequency axis (pulse spectral intensity *S*) and the absorption spectrum of NaI. The sticks represent a sample of 50 ground-state geometries sampled from the ground-state Wigner distribution, with the blue color used to represent geometries in resonance with the pulse.

Having now a mental picture of the Wigner pulse representation and an equation for the promoted nuclear density (eq 8), we can devise PDA—a practical implementation for sampling initial conditions for nonadiabatic dynamics that incorporate implicitly the effect of a laser pulse, in other words, sampling the promoted nuclear density ρ_*p*_^cl^. We start by the usual sampling of the ground-state nuclear probability density ρ_*g*_^cl^(**R**, **P**), producing a set of *N*_*g*_ pairs of nuclear positions and momenta {**R**_*i*_, **P**_*i*_}_*i*=1_^*N*_*g*_^. Different techniques can be used for this task, e.g., the harmonic Wigner sampling^28,29^ (available in most codes for nonadiabatic molecular dynamics) or *ab initio* molecular dynamics with quantum thermostat.^30−32^ Then, excitation energies (*ΔE*_*eg*_(**R**)) and transition dipole moments (μ⃗_*eg*_(**R**)) to the excited electronic state of interest must be obtained for each sampled nuclear configuration **R**_*i*_—this process is also commonly performed to calculate a photoabsorption cross-section from the sampled geometries using the nuclear ensemble approach.^33^ Having collected this information, we now take the first step beyond the standard workflow and randomly select an excitation time *t*′ (from a time window surrounding the laser pulse of interest) and a position-momentum pair {**R**_*i*_, **P**_*i*_}. Using this random excitation time *t*′ and {**R**_*i*_, **P**_*i*_}, a transition probability  can be obtained based on eq 5. This probability is compared to a uniformly generated random number and, if this random number is smaller than the transition probability, the initial condition {**R**_*i*_, **P**_*i*_, *t*′} is accepted. This process of random selection is iterated until a desired number of initial conditions *N*_*p*_ is created {**R**_*j*_, **P**_*j*_, *t*_*j*_^′^}_*j*=1_^*N*_*p*_^ (see Algorithm S1 in the SI for an algorithmic representation of the process).^c^ The excitation time *t*_*j*_^′^ stands for the actual time within the laser pulse envelope when the trajectory is initiated in the excited electronic state with {**R**_*j*_, **P**_*j*_} (in contrast, the vertical sudden approximation would initiate *all* trajectories at the excitation time *t*′ = 0). We note that some {**R**_*i*_, **P**_*i*_} pairs may not be selected at all for excitation, while some other {**R**_*i*_, **P**_*i*_} pairs may get excited at various times during the pulse; in other words, we can assign no or multiple excitation times *t*′ to any given {**R**_*i*_, **P**_*i*_} pair. The algorithm is straightforwardly extended to photoexcitation toward multiple excited electronic states by randomly selecting also the excited state (see the SI). A user-friendly Python implementation of the algorithm is available,^34,35^ and a detailed description of the code is provided in the SI.

Once the initial conditions {**R**_*j*_, **P**_*j*_, *t*_*j*_^′^}_*j*=1_^*N*_*p*_^ are sampled, the trajectory-based nonadiabatic dynamics simulation can be initiated in the electronic state of interest from unique position-momentum pairs {**R**_*j*_, **P**_*j*_} within the initial conditions, starting at time 0. Finally, these resulting simulations should be shifted to their respective excitation times *t*_*j*_^′^ before analysis. We propose to start the simulations at time 0 and then shift them to *t*_*j*_^′^ to avoid repeated calculations of the same simulation just shifted to a different initial time. This way, the unique trajectories are used multiple times by shifting them to different *t*_*j*_^′^ values. Note that the trajectory itself is considered fixed in the ground state until time *t*_*j*_^′^, when it is promoted to the excited electronic state.

We now move to a numerical demonstration of PDA performance. To do so, we propose to compare the results of numerically exact quantum dynamics (QD) simulations of NaI including explicitly a laser pulse to those obtained with FSSH using PDA, that is, incorporating the laser pulse implicitly in the initial conditions as described above.

We start by comparing QD with an explicit laser pulse and FSSH+PDA for the photoexcitation of NaI resulting from three 20 fs laser pulses with different frequencies (Figure 3A). Our comparison is based on the expectation value of the NaI bond length in the first adiabatic excited state *S*_1_, ⟨*R*⟩_*S*_1__, and its standard deviation, ⟨*ΔR*⟩_*S*_1__. FSSH combined with PDA is in excellent agreement with the result obtained by QD with the explicit laser pulse (Figure 3B, note the perfect overlap between dashed and solid lines). Increasing the pulse frequency generates an excited nuclear wavepacket that takes longer to return to the Franck–Condon region following photoexcitation. The 20 fs pulses promote only small portions of the ground-state nuclear density, as seen in Figure 2 depicting the low-frequency pulse. The resulting excited nuclear wavepacket is promoted on different regions of the potential energy curve depending on the laser pulse frequency, each possessing then a different total energy resulting in different periods of oscillations. The violet dashed and solid lines in Figure 3B represent the dynamics obtained from a vertical (or sudden) excitation^d^ of the ground-state nuclear wave function (both in QD and FSSH). While this set of simulations serves as a test of consistency between QD and FSSH (nearly indistinguishable results are obtained), they also clearly spotlight the remarkable differences between the dynamics triggered by the vertical (sudden) excitation and those obtained by considering the effect of a laser pulse. These results make it clear that using different laser pulses triggers excited-state dynamics with distinct time scales and incorporating such effects is crucial for a proper comparison with an experiment: here, the range of oscillation periods varies from 900 to 1500 fs depending on the laser pulse.

**Figure 3:**
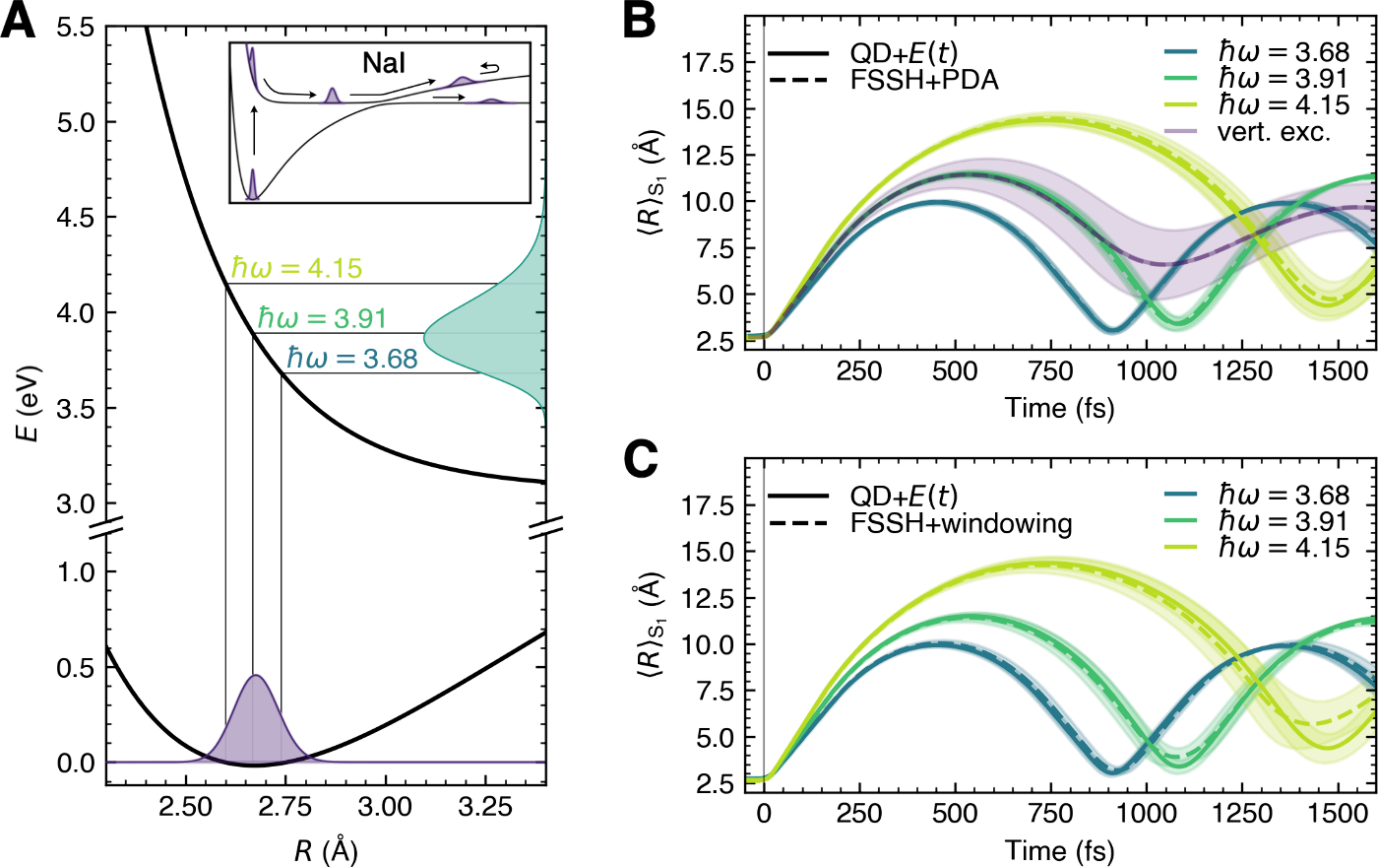
Photoexcitation of NaI with laser pulses of different frequencies, comparing QD with an explicit laser pulse and FSSH with PDA. (A) Potential energy curves of NaI (full range with sketched nuclear wavepacket evolution is given in the inset) alongside the ground-state density (violet) and the absorption spectrum (green) calculated with the nuclear ensemble method. The different frequencies ℏω of the applied laser pulse are depicted. (B) Expectation values of the NaI bond length in *S*_1_ for three different laser pulse frequencies, comparing quantum dynamics with an explicit 20 fs laser pulse (solid lines) and PDA combined with FSSH nonadiabatic dynamics (dashed lines). The shaded area represents  of the nuclear wavepacket (QD) or trajectories (FSSH). Simulations considering vertical excitation are provided as validation. (C) Same as in panel B but for FSSH using a simple windowing approach combined with a time convolution (dashed lines).

When nonadiabatic dynamics simulations account for a laser pulse implicitly, they usually do so in a simple way by imposing an energy window on the selection of initial conditions (symbolized by the vertical dashed lines in Figure 2). Testing this windowing approach reveals that, although less accurate than PDA, it still captures the major effects created by the laser pulse and brings a large improvement over the vertical excitation approach (Figure 3C). The definition of this energy window is, nevertheless, arbitrary. PDA allows us to validate a more rigorous scheme to perform a simple windowing. Considering the properties of the Wigner pulse representation given in eqs 9 and 10, we can approximate it as
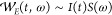
12and recast eq 7 into the following, simplified form
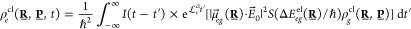
13where the integral has a structure of convolution. Equation 13 provides a clear recipe for a simple windowing and convolution approach that we coined PDAW (promoted density approach for windowing). We can assign a weight
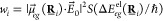
14to each position-momentum pair {**R**_*i*_, **P**_*i*_} from the ground-state sampling. Then, we propagate {**R**_*i*_, **P**_*i*_} in the excited state and calculate an observable  for each trajectory. The total time-dependent observable  is then calculated by summing the weighted (and normalized) observables obtained from each initial condition and convoluting the result with the adequate form of the normalized pulse intensity *I̅*(*t*) = *I*(*t*)/∫_–∞_^∞^ *I*(*t*′) d*t*′:
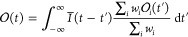
15We emphasize that the weights *w*_*i*_ in eq 15 are based on the pulse spectral intensity *S*(ω) and not the pulse spectrum *Ẽ*(ω) (see eq 14). Applying the PDAW scheme to FSSH for the three Gaussian laser pulses described in Figure 3 outperforms the standard windowing and yields the same results as FSSH combined with PDA, except for dynamics during the pulse as PDAW is based on eq 7 and not eq 8 (see the SI). However, we note that PDAW is still an approximation to PDA and eq 12 will not be valid in general, e.g., for chirped pulses with long duration (see the SI).

How good is PDA to describe pulses of different durations? Let us test the approach for a series of Gaussian pulses with parameters τ = 1, 2.5, 5, 10, and 20 fs considering the central frequency ℏω_0_ = 3.6792 eV (Figure 4). In Figure 4A, we represent the product of the pulse spectral intensity *S* with the ground-state density ρ_*g*_, demonstrating the portions of ρ_*g*_ targeted with the laser pulses of different durations. While the shortest 1 fs laser pulse^e^ encompasses almost the whole ground-state density, the longest 20 fs pulse strikes only a small part of it. Again, PDA combined with FSSH leads to results in excellent agreement with QD using an explicit laser pulse for all pulses tested (Figure 4B), despite the strong effect of the laser pulse on the resulting excited-state dynamics. While the 1 fs pulse leads to a nonadiabatic dynamics closely matching the one obtained after a vertical excitation (depicted in Figure 3B), the 20 fs pulse creates a more confined excited-state nuclear wavepacket (compare shaded areas in Figure 4B). These results once again strongly advocate for including the effect of a laser pulse in nonadiabatic molecular dynamics.

**Figure 4:**
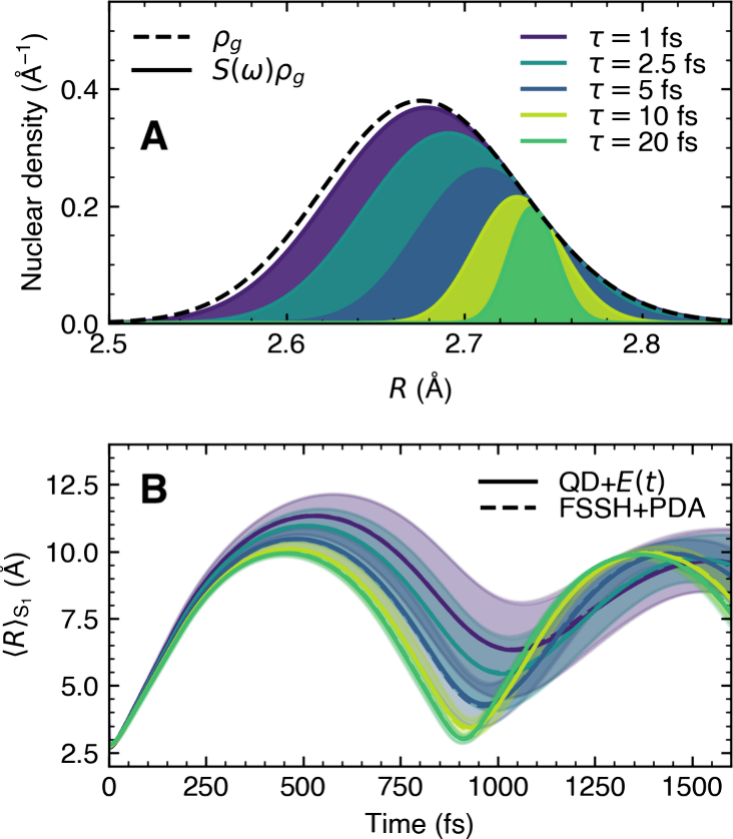
Photoexcitation of NaI with laser pulses of different durations, comparing QD with an explicit laser pulse and FSSH with PDA. (A) The ground-state nuclear density ρ_*g*_ (dashed line) and its product with the spectral intensity *S*(ω) (filled areas) for pulses of different duration τ. (B) Expectation values of the NaI bond length in *S*_1_ for the different laser pulse durations given in (A), all with ℏω_0_ = 3.6792 eV, for QD with an explicit laser pulse (solid lines) and FSSH using PDA (dashed lines). The shaded area represents ⟨*ΔR*⟩_*S*_1__ of the nuclear wavepacket (QD) or trajectories (FSSH). We note that the dashed lines are hardly visible as they almost perfectly overlap the corresponding solid lines.

So far, we have shown that PDA combined with FSSH leads to nonadiabatic dynamics simulation in excellent agreement with quantum dynamics including an explicit laser pulse. Yet we note that the selected cases are all within PDA’s approximations, such as short pulse duration or positive pulse Wigner representation, summarized in the SI. To stress its applicability, we pushed the approach out of its comfort zone in different ways. We briefly summarize here our findings and the interested reader can refer to the SI for the results and comparisons. PDA combined with FSSH provides excellent results even for 100 fs long laser pulse, but disparities with the QD results start to appear for a 500 fs pulse with FSSH. These disparities are not *per se* due to a limitation of PDA for describing such long pulses but are caused by the appearance of quantum-interference effect in the dynamics when the excited-state wavepacket returns to the Franck–Condon region while the interaction with the laser pulse is still going on. We also tested different envelopes for the laser pulse, such as the Lorentzian envelope which exhibits negative regions in its Wigner pulse representation. Both strategies introduced for dealing with negative values—either ignoring them or taking their absolute value—perform well although the agreement is not as perfect as for the Gaussian pulses. Notably, our test simulations show that PDA can also capture excited-state dynamics during the pulse. PDAW proved to be also applicable for excited-state dynamics involving Lorentzian pulses, even if it lacks the dynamical effects observed during the pulse with PDA. Finally, we confirmed the applicability of the PDA strategy for chirped laser pulses.

As a final example, we wish to illustrate the applicability of PDA to a larger molecular system, combined with on-the-fly FSSH dynamics. We selected protonated formaldimine as an example—a molecule well-known to the community and often used for benchmarking nonadiabatic dynamics^36,37^—and simulated its interaction with a Gaussian laser pulse (eq 11, with ω_0_ = 0.40 au and τ = 20 fs). The photodynamics of protonated formaldimine is particularly interesting as the population transfer from the photoexcited *S*_2_ state is notoriously fast. To demonstrate that PDA can also be used as a postprocessing tool for FSSH dynamics, we reuse the 500 position-momentum pairs {**R**_*i*_, **P**_*i*_}_*i*=1_^500^ and corresponding FSSH trajectories from our previous work considering vertical excitation.^36^ From these position-momentum pairs, we generated a set of 50000 initial conditions {**R**_*j*_, **P**_*j*_, *t*_*j*_^′^}_*j*=1_^50000^ using PDA that contains only 63 unique positions and momenta, i.e. only 63 FSSH trajectories are required (see Figure 5). These 63 trajectories were shifted to different initial times *t*_*j*_^′^ creating a new set of 50000 trajectories used for the analysis.

**Figure 5:**
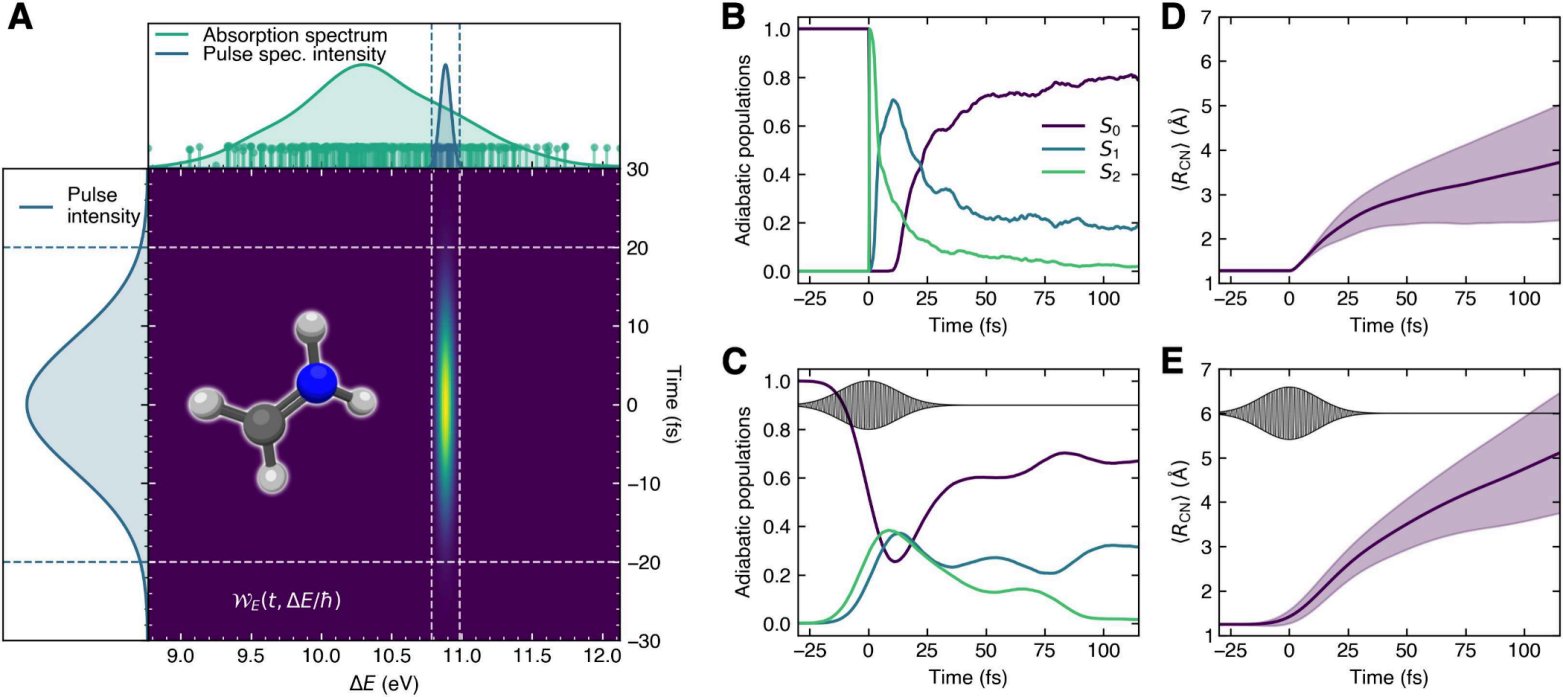
Photodynamics of protonated formaldimine, comparison between FSSH and the vertical (sudden) approximation and FSSH using PDA. (A) The Wigner pulse representation with pulse intensity, spectral intensity, and absorption spectrum of protonated formaldimine. The excitation energies of the 500 nuclear position–momentum pairs are represented as sticks with a height proportional to |μ_*S*_2_*S*_0__(**R**_*i*_)|^2^. Blue sticks are used to depict the pairs that were selected by PDA, while green sticks indicate pairs that were not promoted by the pulse. (B) Time trace of the adiabatic electronic populations for FSSH with a vertical excitation. (C) Time trace of the adiabatic electronic populations for FSSH with PDA. (D) Mean carbon–nitrogen bond length (*R*_CN_) during the FSSH dynamics initiated from a vertical excitation. (E) Mean carbon–nitrogen bond length (*R*_CN_) during the FSSH dynamics initiated from PDA.

The photodynamics of protonated formaldimine is significantly altered by including an implicit laser pulse within PDA. The FSSH simulations with a vertical excitation provide a lifetime for the *S*_2_ electronic state that is comparable to the pulse duration used with PDA. As a result, the population of the *S*_2_ electronic state only reaches a maximum of only 0.4 when the 20 fs laser pulse is included, as the sink of the *S*_2_ population (via nonadiabatic processes) is faster than the source of population from *S*_0_ caused by the laser pulse. The overall population dynamics is also more spread in time within PDA, exemplifying the importance of pulse duration effects on ultrafast processes like internal conversion. The time evolution of the mean bond length (*R*_CN_) is also affected by the finite-energy spectrum of the 20 fs laser pulse, leading to a faster extension in comparison to the FSSH dynamics invoking a vertical excitation. While none of these effects are surprising, they clearly demonstrate the profound consequences of adequately including the effect of a laser pulse in nonadiabatic molecular dynamics. Furthermore, the results highlight that PDA can be used to infer the laser pulse effects in a postprocessing manner from already calculated simulations considering vertical excitation. This can be particularly useful when studying novel molecules for which experiments have yet to be conducted.

In conclusion, we proposed a formalism coined PDA for the implicit inclusion of arbitrary laser pulses in nonadiabatic molecular dynamics at the level of the initial conditions. The core component of this method is the Wigner pulse representation used to calculate an (approximate) promoted nuclear density for the subsequent excited-state dynamics. Sampling the promoted density is the only additional step required in comparison to the standard approach invoking a vertical excitation. We have demonstrated the performance and possible limitations of the formalism by simulating the photoexcitation of NaI with FSSH and PDA using a broad range of laser pulses, leading to excellent agreements with QD simulations including explicitly the laser pulse. The excellent performance of FSSH+PDA contrasts with the poor performance of FSSH when coupled to an explicit laser pulse, as reported in earlier works.^21,22^ We also derived the approximate PDAW formalism that sets the simpler energy windowing and intensity convolution strategy on firm ground. The applicability of the PDA approach to molecular systems (and its use as a postprocessing tool, that is, allowing to test the effect of various laser pulses using the same set of nonadiabatic trajectories) was demonstrated with the photoexcitation of protonated formaldimine. Both PDA and PDAW are implemented in a user-friendly Python code promdens.py available as a Python package in the PyPI repository^34^ or on GitHub^35^ (a full description of the code and its availability is provided in the SI), using as an input only the results of a spectrum calculation obtained with the nuclear ensemble method. The technique is easy to extend to multiple electronic states, yet further developments are needed to describe a coherent superposition of electronic states within the present framework. Overall, the results of our simulations stress the importance of including the laser pulse in nonadiabatic molecular dynamics and the potential dangers of using the vertical (sudden) excitation.

## Supporting Information Available

Derivation of the central equations of the promoted density approximation with definitions of all required quantities; full computational details; extended tests of the methodologies presented in this work (PDA vs PDAW vs windowing, chirped pulses, long laser pulses, Lorentzian envelopes); and description of the PDA code, its input, and its usage (PDF)
Transparent Peer Review report available (PDF)

## Footnotes

a For pulses in the form ε(*t*) cos(ω_0_*t*) the intensity is defined as *I*(*t*) = ^1^/_2_ϵ_0_*cnε*^2^(*t*) where ϵ_0_ is the permittivity, *c* is the speed of light and *n* is the material refractive index.^27^

b We note that defining pulses in the form of an envelope multiplied by an oscillating phase is generally not in line with Maxwell equations and works only for pulses with τ > 2 fs in the visible region and τ > 1.2 fs for UV pulses. More details about the limit of the pulse envelope formulation and its derivation are provided in the SI.

c We use index *j* for the initial conditions generated by PDA, while the index *i* is reserved for the ground-state sampling of position–momentum pairs {**R**_*i*_, **P**_*i*_}.

d Vertical (or sudden) excitation assumes that the whole unchanged ground-state nuclear density is promoted to the excited electronic state instantaneously at time *t*, i.e., ρ_*p*_ = |μ⃗_*eg*_·*E⃗*_0_|^2^δ(*t*)ρ_*g*_.

e A 1 fs laser pulse with a central frequency corresponding to ℏω_0_ = 3.6792 eV is just at the boundary of being valid within a pulse envelope formalism (see the SI).
