# Supplementary material for: Including Photoexcitation Explicitly in Trajectory-Based Nonadiabatic Dynamics at No Cost
Source: J Phys Chem Lett. 2024 Oct 15;15(42):10614–22. doi: 10.1021/acs.jpclett.4c02549 (PMC11514012; doi:10.1021/acs.jpclett.4c02549)
Supplement: Supplementary file 1 — jz4c02549_si_001.pdf [file jz4c02549_si_001.pdf]

Supporting Information: Including photoexcitation explicitly in trajectory-based nonadiabatic dynamics at no cost

Jiří Janoš,[*,†,‡] Petr Slavíček,[*,†] and Basile F. E. Curchod[*,‡]

†*Department of Physical Chemistry, University of Chemistry and Technology, Technická 5, Prague 6, 166 28, Czech Republic*

‡*Centre for Computational Chemistry, School of Chemistry, University of Bristol, Bristol BS8 1TS, United Kingdom*

E-mail: jiri.janos@vscht.cz; petr.slavicek@vscht.cz; basile.curchod@bristol.ac.uk

Contents

1 Intensity, spectrum, and Wigner representation of a laser pulse **S-3**

2 Limits of the laser pulse definition via a pulse envelope **S-6**

3 Derivation of the time-dependent excited-state density **S-8**

4 Algorithm to use the promoted density approach for single and multiple excited states **S-16**

5 Computational details **S-18**

5.1 NaI – sodium iodide S-18

5.2 Protonated formaldimine S-20

5.3 Calculation of observables S-20

5.3.1 Population transfer in QD and PDA S-21

6 Extended tests of PDA and PDAW **S-22**

6.1 Comparing PDA, PDAW, and standard windowing S-22

6.2 Excitation with a chirped pulse: PDA vs. PDAW S-23

6.3 Long laser pulses with PDA S-24

6.4 Lorentzian envelope: negative Wigner probabilities and dynamics during the pulse S-25

7 Python implementation of PDA and PDAW **S-29**

7.1 Installation S-29

7.2 Usage S-29

References **S-32**

1 Intensity, spectrum, and Wigner representation of a laser pulse

In this Section, we aim to review some basic properties of laser pulses and justify facts mentioned in the main text. We start by defining the time-dependent electric field of the laser pulse as $\vec{E}(t) = \vec{E}_0 E(t)$, where $\vec{E}_0$ is the electric field amplitude E_0 multiplied by the polarization vector $\vec{\lambda}$ of the field. In the following, we will focus on the time-dependent complex scalar field $E(t)$,

$$E(t) = \varepsilon(t) \mathrm{e}^{i\gamma(t)}\,, \tag{1}$$

where $\varepsilon(t)$ is an envelope and $\gamma(t)$ is a phase defined as

$$\gamma(t) = \varphi_0 + \omega_0 t + \beta t^2\,, \tag{2}$$

with φ_0 being the carrier-envelope phase, ω_0 the carrier frequency, and β the quadratic phase modulation or linear chirp parameter. The instantaneous frequency of a pulse, i.e., the frequency of the oscillations at every point in time, is then defined as the time derivative of the phase γ,

$$\frac{\mathrm{d}\gamma}{\mathrm{d}t} = \dot{\gamma} = \omega_0 + 2\beta t\,. \tag{3}$$

For unchirped pulses ($\beta = 0$), the instantaneous frequency equals to ω_0. We note that defining the field E as a complex function has the advantage that the spectrum contains only the positive-frequency contributions while defining the field as a real function $E(t) = \varepsilon(t)\cos\gamma(t)$ creates a symmetric spectrum with unphysical negative-frequency components. So while we use the real electric field in the Hamiltonian for the quantum dynamics simulations, we rather work with the complex electric field when deriving its properties.

The intensity of the field, which is the experimentally measured quantity, is defined as

$$I(t) = \frac{1}{2}\epsilon_0 c n \varepsilon^2(t)\,, \tag{4}$$

where ϵ_0 is the permittivity, c is the speed of light and n is the material refractive index.[S1]

The spectrum of the field comes from the Fourier transform of E,

$$\tilde{E}(\omega) = \mathcal{F}[E(t)] = \int_{-\infty}^{\infty} E(t)\mathrm{e}^{-i\omega t}\,\mathrm{d}t = |\tilde{E}(\omega)|\mathrm{e}^{i\phi(\omega)}\,, \tag{5}$$

where $|\tilde{E}(\omega)|$ denotes the spectral amplitude and $\phi(\omega)$ spectral phase. However, the experimentally accessible quantity is rather the spectral intensity $S(\omega)$ than the spectrum. The spectral intensity can be derived as

$$S(\omega) = \frac{\epsilon_0 c n}{\pi}\left|\tilde{E}(\omega)\right|^2 \tag{6}$$

and equals to the square of the pulse spectrum $\tilde{E}$ multiplied by constants.[S1]

Let us now comment on the parameters used to determine and report pulses. The ex-

perimentally accessible quantities are the intensities $I(t)$ and $S(\omega)$. Therefore, the full width at half maximum (FWHM) values for the temporal resolution (τ) and the spectral resolution (Ω) are determined from the respective intensities. On the other hand, theoreticians are usually accustomed to working with the electric field $E(t)$ and pulse spectrum $\tilde{E}(\omega)$ and report the FWHM quantities for them – this disparity often leads to a misunderstanding between theoreticians and experimentalists. The way one defines the FWHM parameters is arbitrary, but we need to remain consistent. In this work, we advocate the use of the FWHM parameters for intensities. Not only are they directly provided in experiments but intensities also determine the temporal convolution of theoretical quantities and the energy windowing, as we will show later. In practice, this means that when we set the parameter τ, it is for the FWHM of the intensity $I(t) \approx \varepsilon^2(t)$ and not the field envelope $\varepsilon(t)$. The FWHM parameter of the envelope $\varepsilon(t)$ depends on the specific envelope form and is easy to convert.

In analogy to quantum mechanics, where we can represent the wavefunction in either position or momentum space but never in both simultaneously, we cannot directly represent the field in the time and frequency domains together due to their Fourier transform relationship. Nevertheless, we can borrow the concept of Wigner representation from quantum mechanics, which allows us to construct phase-space quantities, for laser pulses. The Wigner representation $\mathcal{W}_E$ of a laser pulse defined by $E(t)$ reads[S1]

$$\mathcal{W}_E(t,\omega) = \int_{-\infty}^{\infty} E\left(t + \frac{s}{2}\right) E^*\left(t - \frac{s}{2}\right) \mathrm{e}^{-i\omega s}\,\mathrm{d}s \tag{7}$$

$$= \frac{1}{2\pi}\int_{-\infty}^{\infty} \tilde{E}\left(\omega + \frac{s}{2}\right) \tilde{E}^*\left(\omega - \frac{s}{2}\right) \mathrm{e}^{its}\,\mathrm{d}s \tag{8}$$

and presents a simultaneous representation of the laser pulse in the time and frequency domain.

However, we need to approach the concept of a Wigner representation for laser pulses with the same care as in quantum mechanics. The Wigner representation of a laser pulse allows us to plot the correlation between the temporal and spectral domains, which is useful especially for chirped pulses, but one should not think about it as a physical object. For example, we cannot interpret the Wigner representation as a probability density of a certain frequency at a given time because $\mathcal{W}_E$ can acquire negative values. Measurable physical quantities can be obtained from integrated Wigner distribution: if we integrate $\mathcal{W}_E$ over the frequency domain,

$$\int_{-\infty}^{\infty} \mathcal{W}_E(t,\omega)\,\mathrm{d}\omega = \int_{-\infty}^{\infty}\int_{-\infty}^{\infty} E\left(t + \frac{s}{2}\right) E^*\left(t - \frac{s}{2}\right) \mathrm{e}^{-i\omega s}\,\mathrm{d}s\,\mathrm{d}\omega \tag{9}$$

$$= \int_{-\infty}^{\infty} E\left(t + \frac{s}{2}\right) E^*\left(t - \frac{s}{2}\right) \int_{-\infty}^{\infty} \mathrm{e}^{-i\omega s}\,\mathrm{d}\omega\,\mathrm{d}s \tag{10}$$

$$= 2\pi \int_{-\infty}^{\infty} E\left(t + \frac{s}{2}\right) E^*\left(t - \frac{s}{2}\right) \delta(s)\,\mathrm{d}s \tag{11}$$

$$= 2\pi\,|E(t)|^2 = 2\pi\varepsilon(t)^2 \approx I(t)\,, \tag{12}$$

we get the square of the field envelope which is proportional to the intensity $I(t)$. We used

the identity $\int_{-\infty}^{\infty} \mathrm{e}^{-i\omega s}\,\mathrm{d}\omega = 2\pi\delta(s)$. On the other hand, if we integrate over the time domain using the definition Eq. (8),

$$\int_{-\infty}^{\infty} \mathcal{W}_E(t,\omega)\,\mathrm{d}t = \frac{1}{2\pi}\int_{-\infty}^{\infty}\int_{-\infty}^{\infty} \tilde{E}\left(\omega+\frac{s}{2}\right)\tilde{E}^*\left(\omega-\frac{s}{2}\right)\mathrm{e}^{its}\,\mathrm{d}s\,\mathrm{d}t \tag{13}$$

$$= \frac{1}{2\pi}\int_{-\infty}^{\infty} \tilde{E}\left(\omega+\frac{s}{2}\right)\tilde{E}^*\left(\omega-\frac{s}{2}\right)\int_{-\infty}^{\infty}\mathrm{e}^{its}\,\mathrm{d}t\,\mathrm{d}s \tag{14}$$

$$= \int_{-\infty}^{\infty} \tilde{E}\left(\omega+\frac{s}{2}\right)\tilde{E}^*\left(\omega-\frac{s}{2}\right)\delta(s)\,\mathrm{d}s \tag{15}$$

$$= \left|\tilde{E}(\omega)\right|^2 \approx S(\omega)\,, \tag{16}$$

we obtain the square of the pulse spectrum which is proportional to the spectral intensity $S(\omega)$. Similarly, various moments of the field, the instantaneous frequency, and other quantities can be obtained, for which we refer the reader to Ref. S1.

The Wigner pulse representation $\mathcal{W}_E$ can be recast into a simpler form if the pulse is defined in terms of an envelope ε and an oscillating phase γ (see Eq. (1)). Substituting Eq. (1) into Eq. (7) leads to

$$\mathcal{W}_E(t,\omega) = \int_{-\infty}^{\infty} \varepsilon\left(t+\frac{s}{2}\right)\mathrm{e}^{i\gamma(t+s/2)}\varepsilon^*\left(t-\frac{s}{2}\right)\mathrm{e}^{-i\gamma(t-s/2)}\mathrm{e}^{-i\omega s}\,\mathrm{d}s \tag{17}$$

$$= \int_{-\infty}^{\infty} \varepsilon\left(t+\frac{s}{2}\right)\varepsilon^*\left(t-\frac{s}{2}\right)\mathrm{e}^{-i[\omega s+\gamma(t-s/2)-\gamma(t+s/2)]}\,\mathrm{d}s \tag{18}$$

$$= \int_{-\infty}^{\infty} \varepsilon\left(t+\frac{s}{2}\right)\varepsilon^*\left(t-\frac{s}{2}\right)\mathrm{e}^{-i[\omega-\dot{\gamma}(t)]s}\,\mathrm{d}s\,, \tag{19}$$

where we employed a Taylor expansion to derive $\gamma(t-s/2)-\gamma(t+s/2) = -(\omega_0+2\beta t)s = -\dot{\gamma}(t)s$. The time derivative of the phase $\dot{\gamma}$ stands for the instantaneous frequency, see Eq. (3). We can now define a Wigner representation of the pulse envelope

$$\mathcal{W}_\varepsilon(t,\omega) = \int_{-\infty}^{\infty} \varepsilon\left(t+\frac{s}{2}\right)\varepsilon^*\left(t-\frac{s}{2}\right)\mathrm{e}^{-i\omega s}\,\mathrm{d}s\,. \tag{20}$$

Note that the complex conjugation is not necessary for real envelopes yet we keep it to highlight the analogy to $\mathcal{W}_E$. Following from Eq. (19), $\mathcal{W}_E$ and $\mathcal{W}_\varepsilon$ are connected via a simple relation:

$$\mathcal{W}_E(t,\omega) = \mathcal{W}_\varepsilon(t,\omega-\dot{\gamma})\,. \tag{21}$$

The concept of $\mathcal{W}_\varepsilon$ is less general than $\mathcal{W}_E$, as it works only for pulses defined as in Eq. (1) and is not suitable for ultrashort pulses (see Section 2). Yet, it is more efficient for numerical implementations and is utilized in our Python code (described in Section 7).

2 Limits of the laser pulse definition via a pulse envelope

Through this work, we represent laser pulses in the form of an envelope times an oscillating field, as in Eq. (1). Although it is the most often used representation of a laser pulse, care is needed when using it. The following condition for a laser pulse in free space must hold

$$\int_{-\infty}^{\infty} E(t)\,\mathrm{d}t = 0 \tag{22}$$

to fulfill Maxwell equations. Several reasons for this condition to hold are comprehensively presented in Ref. S2. We shall mention only one of them here: *if the integral over the electric field is not equal to zero, a direct current is induced, which is unphysical in free space*.

Whether a laser pulse is physical and fulfills the condition provided by Eq. (22) can be easily verified from the zero frequency component of the pulse spectrum $\tilde{E}$:

$$\int_{-\infty}^{\infty} E(t)\,\mathrm{d}t = \int_{-\infty}^{\infty} E(t)\mathrm{e}^{i(\omega=0)t}\,\mathrm{d}t = \tilde{E}(0) = 0\,. \tag{23}$$

Thus, if a given definition of a laser pulse exhibits a nonzero spectrum at zero frequency, this laser pulse is not physical. As such, Eq. (23) provides a quick check of whether a laser pulse definition is physical or not.

While for longer pulses (more than a few femtoseconds), the definition of a laser pulse given by Eq. (1) is valid based on the condition given in Eq. (22), ultrashort pulses shorter than a femtoseconds approach the physical limits of such a representation. To further highlight this limitation, we have estimated the range of validity of laser pulses defined with a Gaussian

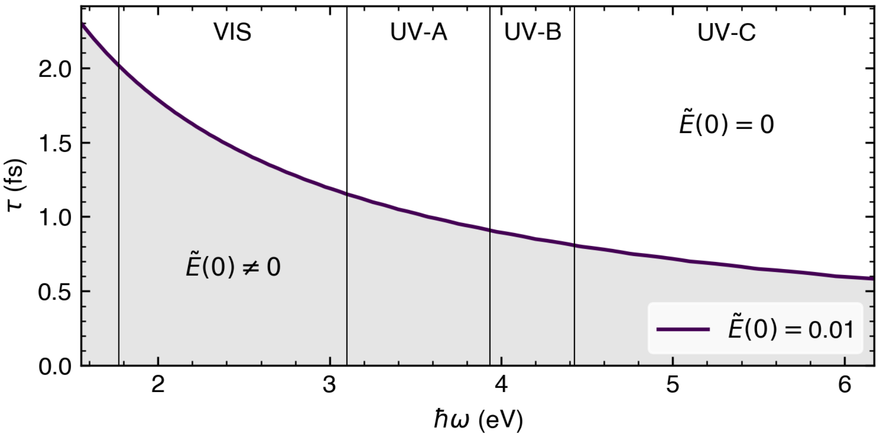

Figure S1: Validity of the Gaussian envelope representation for laser pulses. For each field frequency ω, we calculated the envelope FWHM parameter (τ) at which the normalized (maximum equal to 1) pulse spectrum has a value of 0.01 at zero frequency, i.e., $\tilde{E}(0) = 0.01$. If the value of $\tilde{E}(0)$ is less than 0.01, we consider that the condition given by Eq. (22) is fulfilled. If the value of $\tilde{E}(0)$ is bigger than 0.01, we consider that the pulse is unphysical. The fit of the curve with an inverse function yields $\tau[\mathrm{fs}] = 3.5730048296/\hbar\omega[\mathrm{eV}]$.

envelope, $\varepsilon(t) = \exp\left(-2\ln 2\frac{t^2}{\tau^2}\right)$ (see Figure S1). Based on Figure S1, we propose a rule of thumb for estimating whether a laser pulse can or cannot be represented as a Gaussian envelope time an oscillating field: *If τ (in femtoseconds) for the Gaussian envelope is larger than* 3.6 *divided by $\hbar\omega$ (in electronvolts), the pulse is physical and fulfills Maxwell equations.* Expressed for different wavelengths of light: the τ parameter should be bigger than 2 fs in the visible range and larger than 1.2 fs for UV pulses. Note that for a 2 fs pulse, the spectral bandwidth Ω is roughly 1 eV, and for a 1.2 fs pulse it is about 1.5 eV. Such bandwidths are broad enough to encompass the typical absorption bands of a molecule, making the vertical excitation approximation well justified for such short pulses.

We note that we can easily define pulses fulfilling the condition of Eq. (22) by using the vector potential $\vec{A}(t) = \vec{A}_0 A(t)$, where $\vec{A}_0$ is the vector potential amplitude A_0 multiplied by the polarization vector $\vec{\lambda}$. The electric field is defined from the vector potential as

$$\vec{E}(t) = -\frac{1}{c}\frac{\mathrm{d}\vec{A}(t)}{\mathrm{d}t} = -\frac{1}{c}\vec{A}_0\frac{\mathrm{d}A(t)}{\mathrm{d}t} = \vec{A}_0 E(t)\,. \tag{24}$$

Thus, any definition of $A(t)$ that gives a value of zero at the limits of $t = +\infty$ and $t = -\infty$ will create an electric field fulfilling the condition of Eq. (22):

$$\int_{-\infty}^{\infty} E(t)\,\mathrm{d}t = -\frac{1}{c}\int_{-\infty}^{\infty}\frac{\mathrm{d}A(t)}{\mathrm{d}t}\,\mathrm{d}t = -\frac{1}{c}\left[\lim_{t\to\infty} A(t) - \lim_{t\to-\infty} A(t)\right] = 0\,. \tag{25}$$

Returning to pulses defined by a pulse envelope, one can consider a vector potential in the following form

$$A(t) = -\frac{c}{\omega}\varepsilon(t)\sin(\omega t)\,. \tag{26}$$

The scalar electric field obtained from such a definition reads

$$E(t) = -\frac{1}{c}\frac{\mathrm{d}A(t)}{\mathrm{d}t} = \varepsilon(t)\cos(\omega t) + \frac{1}{\omega}\dot{\varepsilon}(t)\sin(\omega t)\,, \tag{27}$$

where the first term, $\varepsilon(t)\cos(\omega t)$, is the standard envelope times oscillating phase (real in this case) while the second term, $\frac{1}{\omega}\dot{\varepsilon}(t)\sin(\omega t)$, acts like a correction ensuring that the condition of Eq. (22) is fulfilled. Hence, we can define even ultrashort pulses in terms of envelopes, as long as we do so for the vector potential and work with the corresponding correction term. To connect the present discussion to the topics of the previous chapter, one would have to calculate the Wigner transformation from the full electric field formalism, i.e., calculate $\mathcal{W}_E$, and not just from the pulse envelope formalism ($\mathcal{W}_\varepsilon$). Finally, the equations above connect our work with the work of Martínez-Mesa and Saalfrank[S3] who derived their equation for excited-state density in terms of $\mathcal{W}_\varepsilon$.

3 Derivation of the time-dependent excited-state density

In this Section, we outline a complete derivation of the time-dependent excited-state density formula as presented in Eq. (8) in the main article. The original fundamental work on this topic was published by Li, Fang, and Martens[S4] creating a framework for calculating pump-probe spectra. The work was then extended by Shen and Cina[S5] who alleviated some of the approximations introduced previously. While both works derived their leading formula in the density matrix formalism using the quantum Liouville equation, a similar formula was derived by Meier and Engel[S6] in 2002 from a wavefunction perspective rather than the density matrix perspective, avoiding problems with continuum states. Yet, all the aforementioned works considered a specific form of the laser envelope, usually a Gaussian one. The generalization to an arbitrary pulse envelope was achieved by Martínez-Mesa and Saalfrank,[S3] who realized that the interaction can be expressed as the pulse envelope Wigner transform $\mathcal{W}_\varepsilon$. Although this generalization brings some flexibility, it still relies on representing the pulse as an envelope times an oscillating phase, which may not always be justifiable for ultrashort pulses (see Section 2). Furthermore, none of the works discussed above-derived equations considering the dependence of the transition dipole moment on nuclear positions (but instead considered it as constant). In the following paragraphs, we derive an equation for the excited-state density following a path similar to Meier and Engel, yet for a general electric field. Inspired by Martínez-Mesa and Saalfrank, we express the laser pulse in the Wigner representation, but without any restriction on its form – making our formulation compatible with ultrashort pulses. We will show how the pulse envelope formulation appears from our formalism, offering a connection with the work of Martínez-Mesa and Saalfrank. We also generalize the formalism to position-dependent transition dipole moments. We finally propose a detailed discussion on the underlying assumptions of the strategy and the validity of its approximations.

Let us consider a molecular system with two electronic states characterized by the (stationary) electronic wavefunctions ϕ_g (ground state) and ϕ_e (excited state), as well as time-dependent ground-state ($\psi_g(t)$) and excited-state ($\psi_e(t)$) nuclear wavefunctions. The total nuclear wavefunction for the system reads

$$|\Psi(t)\rangle = \begin{pmatrix} \psi_g(t) \\ \psi_e(t) \end{pmatrix} . \tag{28}$$

The Hamiltonian for our system, expressed in the basis of the two electronic states, can be separated into a time-independent Hamiltonian $\mathbf{H}_0$ and a small interaction term $\mathbf{V}_{\text{int}}$ as is typical in perturbation theory,

$$\mathbf{H} = \mathbf{H}_0 + \mathbf{V}_{\text{int}} = \begin{pmatrix} \hat{H}_g & 0 \\ 0 & \hat{H}_e \end{pmatrix} + \begin{pmatrix} 0 & \hat{V}_{\text{int}} \\ \hat{V}_{\text{int}} & 0 \end{pmatrix} . \tag{29}$$

The components of the time-independent Hamiltonian,

$$\hat{H}_g(\underline{\mathbf{R}}) = \hat{T} + E_g^{\text{el}}(\underline{\mathbf{R}}) \tag{30}$$

$$\hat{H}_e(\underline{\mathbf{R}}) = \hat{T} + E_e^{\text{el}}(\underline{\mathbf{R}}) \, , \tag{31}$$

depend on the nuclear kinetic energy operator $\hat{T}$ and the electronic energies $E^{\mathrm{el}}_{g/e}(\underline{\mathbf{R}})$, where $\underline{\mathbf{R}}$ is the the nuclear position vector. Note that the derivation is performed in the position representation; therefore, operators depending only on $\underline{\mathbf{R}}$ do not bear the $\wedge$ in our notation. The time-dependent interaction of the molecule with the electric field is defined within the dipole approximation as

$$\hat{V}_{\mathrm{int}}(\underline{\mathbf{R}}, t) = -\vec{\mu}_{eg}(\underline{\mathbf{R}}) \cdot \vec{E}_0 E(t) \, , \tag{32}$$

with $\vec{\mu}_{eg}(\underline{\mathbf{R}})$ denoting the position-dependent transition dipole moment $\langle \phi_e | \hat{\mu} | \phi_g \rangle$, $\vec{E}_0$ standing for the laser electric field amplitude, and $E(t)$ being a real time-dependent electric field. From now on, we will not specifically emphasize the dependence of $\hat{H}_{g/e}$, $E^{\mathrm{el}}_{g/e}$, $\hat{V}_{\mathrm{int}}$, and $\vec{\mu}_{eg}$ on the nuclear positions $\underline{\mathbf{R}}$ for the sake of simplicity, yet it needs to be borne in mind during the derivation.

We want to solve the time-dependent Schrödinger equation,

$$i\hbar \frac{\mathrm{d}|\Psi(t)\rangle}{\mathrm{d}t} = (\mathbf{H}_0 + \mathbf{V}_{\mathrm{int}}) \, |\Psi(t)\rangle \, , \tag{33}$$

in terms of first-order perturbation theory, where the nuclear wavefunction is defined through the zero-order and first-order wavefunctions $|\Psi\rangle = |\Psi^{(0)}\rangle + |\Psi^{(1)}\rangle$. Considering the initial conditions $\psi_g^{(1)}(t_0) = \psi_e^{(1)}(t_0) = \psi_e^{(0)}(t_0) = 0$, and $\psi_g^{(0)}(t_0) = \psi_g$, the working equations read

$$|\Psi^{(0)}(t)\rangle = \mathrm{e}^{-\frac{i}{\hbar}\mathbf{H}_0(t-t_0)} |\Psi(t_0)\rangle \tag{34}$$

$$|\Psi^{(1)}(t)\rangle = -\frac{i}{\hbar} \mathrm{e}^{-\frac{i}{\hbar}\mathbf{H}_0 t} \int_{t_0}^{t} \mathrm{e}^{\frac{i}{\hbar}\mathbf{H}_0 t'} \mathbf{V}_{\mathrm{int}}(t') |\Psi^{(0)}(t')\rangle \, \mathrm{d}t' \, . \tag{35}$$

Solving the equations for the excited-state nuclear wavefunction leads to

$$\psi_e(t) = -\frac{i}{\hbar} \mathrm{e}^{-\frac{i}{\hbar}\hat{H}_e t} \int_{t_0}^{t} \mathrm{e}^{\frac{i}{\hbar}\hat{H}_e t'} \hat{V}_{\mathrm{int}}(t') \mathrm{e}^{-\frac{i}{\hbar}\hat{H}_g (t'-t_0)} \psi_g \, \mathrm{d}t' \, . \tag{36}$$

Reading the equation from the right, the initial ground-state nuclear wavefunction ψ_g is propagated in the ground electronic state from time t_0 to t', at time t' it interacts with the laser pulse and gets promoted to the excited electronic state, before being (back) propagated to time 0.[a] This is done for all times between t and t_0 yielding what we will call a time-zero excited-state wavefunction ψ_z,

$$\psi_z(t) = \int_{t_0}^{t} \mathrm{e}^{\frac{i}{\hbar}\hat{H}_e t'} \hat{V}_{\mathrm{int}} \mathrm{e}^{-\frac{i}{\hbar}\hat{H}_g (t'-t_0)} \psi_g \, \mathrm{d}t' \, . \tag{37}$$

The wavefunction ψ_z is constructed such that, if propagated in the excited state from time

[a] To clarify, the propagation does not go from t' back to time t_0 but to the time zero ($t = 0$). Nevertheless, we could backpropagate to an arbitrary time given we would then propagate from this arbitrary time back to the time t. In the end, the operator acting is $\mathrm{e}^{-\frac{i}{\hbar}\hat{H}_e(t-t')}$. For convenience in our derivation, we work with time 0.

0 to time t, it yields the correct excited-state wavefunction $\psi_e(t)$,

$$\psi_e(t) = -\frac{i}{\hbar} \mathrm{e}^{-\frac{i}{\hbar}\hat{H}_e t} \psi_z \,. \tag{38}$$

The wavefunction ψ_z is generally time-dependent since the upper integration limit depends on t. However, if time t is a time after the pulse, it becomes a stationary (time-independent) wavefunction. Therefore, ψ_z can be viewed as the initial condition already incorporating the laser pulse that only needs to be propagated in the excited state. However, to obtain a useful formulation for trajectory-based techniques, we need to articulate this equation in terms of densities rather than wavefunctions.

The excited-state density $\rho_e(t) = |\psi_e(t)\rangle\langle\psi_e(t)|$ can be written in terms of time-zero excited-state density $\rho_z(t) = |\psi_z\rangle\langle\psi_z|$ and the quantum Liouville operator in the excited state $\mathcal{L}_e$,

$$\rho_e(t) = |\psi_e\rangle\langle\psi_e| = \frac{1}{\hbar^2} \mathrm{e}^{-\frac{i}{\hbar}\hat{H}_e t} |\psi_z\rangle\langle\psi_z| \mathrm{e}^{\frac{i}{\hbar}\hat{H}_e t} = \frac{1}{\hbar^2} \mathrm{e}^{\mathcal{L}_e t} \rho_z(t) \,. \tag{39}$$

From now on, we focus only on the dynamics after the laser pulse. Thus, we set the integration limits in Eq. (37) such that they encompass the whole pulse, making ρ_z time-independent. Since the interaction is zero when the laser pulse is over, we can integrate from $-\infty$ to ∞ without any loss of generality for dynamics after the pulse. Eq. (39) is then valid only for times t after the laser pulse. The time-independent density ρ_z now takes the form

$$\rho_z = \int_{-\infty}^{\infty}\int_{-\infty}^{\infty} \mathrm{e}^{\frac{i}{\hbar}\hat{H}_e \tau'} \hat{V}_{\mathrm{int}}(\tau') \mathrm{e}^{-\frac{i}{\hbar}\hat{H}_g \tau'} |\psi_g\rangle\langle\psi_g| \mathrm{e}^{\frac{i}{\hbar}\hat{H}_g \tau} \hat{V}^*_{\mathrm{int}}(\tau) \mathrm{e}^{-\frac{i}{\hbar}\hat{H}_e \tau} \,\mathrm{d}\tau' \,\mathrm{d}\tau \,. \tag{40}$$

We now consider that the initial ground-state density ρ_g is an eigenfunction of the time-independent ground-state Hamiltonian $\hat{H}_g$.[b] Substituting $\tau = t' + \frac{s}{2}$ and $\tau' = t' - \frac{s}{2}$, we get

$$\rho_z = \int_{-\infty}^{\infty} \mathrm{e}^{\frac{i}{\hbar}\hat{H}_e t'} \left[\int_{-\infty}^{\infty} \mathrm{e}^{-\frac{i}{\hbar}\hat{H}_e \frac{s}{2}} \hat{V}_{\mathrm{int}}\left(t' - \frac{s}{2}\right) \mathrm{e}^{\frac{i}{\hbar}\hat{H}_g \frac{s}{2}} \rho_g \mathrm{e}^{\frac{i}{\hbar}\hat{H}_g \frac{s}{2}} \hat{V}^*_{\mathrm{int}}\left(t' + \frac{s}{2}\right) \mathrm{e}^{-\frac{i}{\hbar}\hat{H}_e \frac{s}{2}} \,\mathrm{d}s \right] \mathrm{e}^{-\frac{i}{\hbar}\hat{H}_e t'} \,\mathrm{d}t' \tag{41}$$

$$= \int_{-\infty}^{\infty} \mathrm{e}^{\frac{i}{\hbar}\hat{H}_e t'} \rho_p(t') \mathrm{e}^{-\frac{i}{\hbar}\hat{H}_e t'} \,\mathrm{d}t' = \int_{-\infty}^{\infty} \mathrm{e}^{-\mathcal{L}_e t'} \rho_p(t') \,\mathrm{d}t' \,, \tag{42}$$

where $\mathrm{e}^{-\mathcal{L}_e t'}$ is a backward propagator from time t' to time 0 and what we denoted as a promoted density ρ_p is defined as

$$\rho_p(t') = \int_{-\infty}^{\infty} \mathrm{e}^{-\frac{i}{\hbar}\hat{H}_e \frac{s}{2}} \hat{V}_{\mathrm{int}}\left(t' - \frac{s}{2}\right) \mathrm{e}^{\frac{i}{\hbar}\hat{H}_g \frac{s}{2}} \rho_g \mathrm{e}^{\frac{i}{\hbar}\hat{H}_g \frac{s}{2}} \hat{V}^*_{\mathrm{int}}\left(t' + \frac{s}{2}\right) \mathrm{e}^{-\frac{i}{\hbar}\hat{H}_e \frac{s}{2}} \,\mathrm{d}s \,. \tag{43}$$

The distinction between ρ_z and ρ_p and their interpretation are represented graphically in Fig. S2.

[b] This means that ρ_g is stationary under $\hat{H}_g$ and that $\mathrm{e}^{-\frac{i}{\hbar}\hat{H}_g t'}|\psi_g\rangle = \mathrm{e}^{-\frac{i}{\hbar}E_g^0 t'}|\psi_g\rangle$.

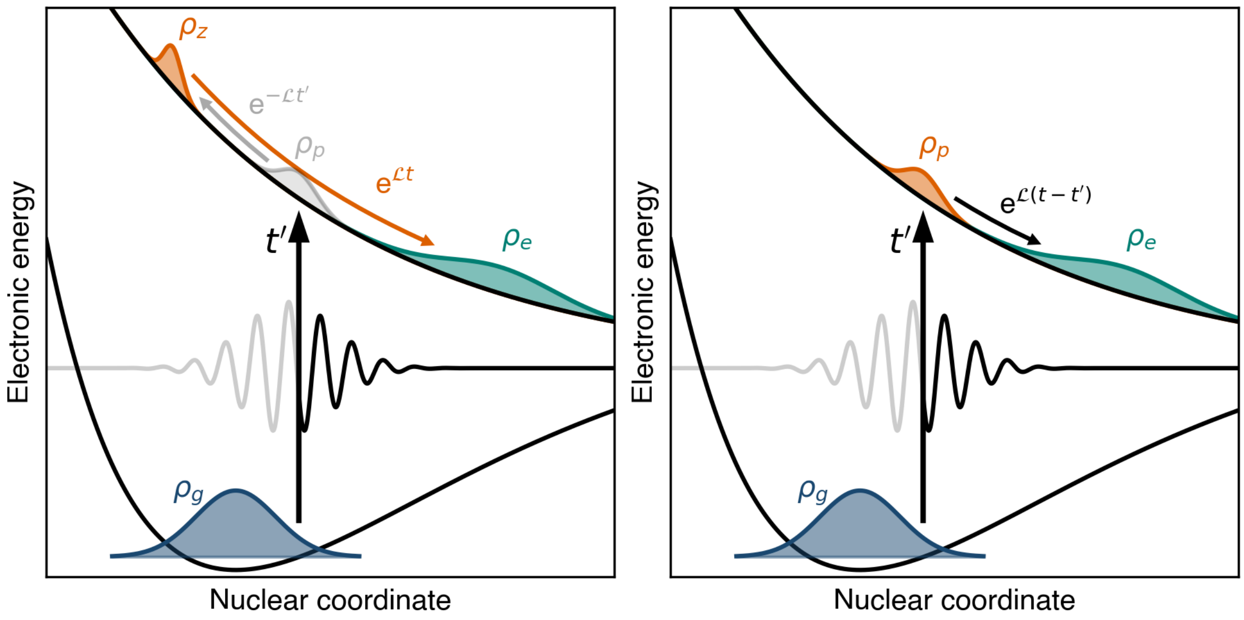

Figure S2: Schematic representation of the photoexcitation process and subsequent excited-state dynamics expressed in terms of the time-zero density ρ_z and the promoted density ρ_p (left), as well as only the promoted density ρ_p (right). Both pictures lead to the same excited-state density ρ_e.

Substituting the definition of interaction potential (Eq. (32)) into Eq. (43), we get

$$\rho_p(t') = \int_{-\infty}^{\infty} E\left(t' + \frac{s}{2}\right) E\left(t' - \frac{s}{2}\right) e^{-\frac{i}{\hbar}\hat{H}_e\frac{s}{2}} \left[\vec{\mu}_{eg} \cdot \vec{E}_0\right] e^{\frac{i}{\hbar}\hat{H}_g\frac{s}{2}} \rho_g e^{\frac{i}{\hbar}\hat{H}_g\frac{s}{2}} \left[\vec{\mu}_{eg} \cdot \vec{E}_0\right] e^{-\frac{i}{\hbar}\hat{H}_e\frac{s}{2}} \mathrm{d}s\,, \tag{44}$$

where we consider the electric field as a real function, i.e., $E^*(t) = E(t)$. The task is now to deal with the operator $e^{-\frac{i}{\hbar}\hat{H}_e\frac{s}{2}} \left[\vec{\mu}_{eg} \cdot \vec{E}_0\right] e^{\frac{i}{\hbar}\hat{H}_g\frac{s}{2}}$. If we consider that the transition dipole moment is a constant, we can take advantage of the Baker–Campbell–Hausdorff (BCH) formula ($e^{\hat{A}}e^{\hat{B}} = e^{\hat{A}+\hat{B}+\frac{1}{2}[\hat{A},\hat{B}]+\cdots}$) to its first order, and express the operator as an exponential of the electronic energy difference

$$e^{-\frac{i}{\hbar}\hat{H}_e\frac{s}{2}} \left[\vec{\mu}_{eg} \cdot \vec{E}_0\right] e^{\frac{i}{\hbar}\hat{H}_g\frac{s}{2}} = \vec{\mu}_{eg} \cdot \vec{E}_0 e^{-\frac{i}{\hbar}\hat{H}_e\frac{s}{2}} e^{\frac{i}{\hbar}\hat{H}_g\frac{s}{2}} \overset{\text{BCH}}{\approx} \vec{\mu}_{eg} \cdot \vec{E}_0 e^{-\frac{i}{\hbar}(E_e^{\text{el}} - E_g^{\text{el}})\frac{s}{2}}\,. \tag{45}$$

While this approach is convenient, we decided here to retain the dependence of the transition dipole moment on the nuclear coordinates and instead to apply the first-order Baker–

Campbell–Hausdorff formula twice

$$e^{-\frac{i}{\hbar}\hat{H}_e\frac{s}{2}}\left[\vec{\mu}_{eg}\cdot\vec{E}_0\right]e^{\frac{i}{\hbar}\hat{H}_g\frac{s}{2}} = e^{-\frac{i}{\hbar}\hat{H}_e\frac{s}{2}}e^{\ln(\vec{\mu}_{eg}\cdot\vec{E}_0)}e^{\frac{i}{\hbar}\hat{H}_g\frac{s}{2}} \tag{46}$$

$$\overset{\text{BCH}}{\approx} e^{-\frac{i}{\hbar}\hat{H}_e\frac{s}{2}+\ln(\vec{\mu}_{eg}\cdot\vec{E}_0)}e^{\frac{i}{\hbar}\hat{H}_g\frac{s}{2}} \tag{47}$$

$$\overset{\text{BCH}}{\approx} e^{-\frac{i}{\hbar}(\hat{H}_e-\hat{H}_g)\frac{s}{2}+\ln(\vec{\mu}_{eg}\cdot\vec{E}_0)} \tag{48}$$

$$= e^{-\frac{i}{\hbar}(E_e^{\text{el}}-E_g^{\text{el}})\frac{s}{2}+\ln(\vec{\mu}_{eg}\cdot\vec{E}_0)} = \vec{\mu}_{eg}\cdot\vec{E}_0 e^{-\frac{i}{\hbar}\Delta E_{eg}^{\text{el}}\frac{s}{2}}\,. \tag{49}$$

These approximations are in general valid for short times (meaning that the laser pulse should be of a short duration) and slowly varying $\vec{\mu}_{eg}$ and $\Delta E_{eg}^{\text{el}}$. The limits of these approximations will be studied at the end of this Section, with all the other approximations and assumptions made throughout the derivation. Taking Eq. (49) and inserting it into Eq. (44) leads to

$$\rho_p(t') = |\vec{\mu}_{eg}\cdot\vec{E}_0|^2\int_{-\infty}^{\infty} E(t'+\frac{s}{2})E(t'-\frac{s}{2})e^{-\frac{i}{\hbar}\Delta E_{eg}^{\text{el}}s}\,\mathrm{d}s\rho_g \tag{50}$$

$$= |\vec{\mu}_{eg}\cdot\vec{E}_0|^2\mathcal{W}_E(t',\Delta E_{eg}^{\text{el}}/\hbar)\rho_g\,, \tag{51}$$

where we have identified the Wigner representation of the pulse $\mathcal{W}_E$ as defined in Eq. (7). Our Eq. (51) for a general form of laser pulses can be also written in terms of the pulse envelope Wigner transform $\mathcal{W}_\varepsilon$, as introduced by Martínez-Mesa and Saalfrank,[S3] using the identity defined in Eq. (21), leading to

$$\rho_p(t') = |\vec{\mu}_{eg}\cdot\vec{E}_0|^2\mathcal{W}_\varepsilon(t',\Delta E_{eg}^{\text{el}}/\hbar-\dot{\gamma})\rho_g\,, \tag{52}$$

which is designed for laser pulses defined as an envelope times an oscillating phase (see Eq. (1)).

The time-dependent excited-state density ρ_e from Eq. (39), combined with Eq. (42) and Eq. (51), now reads

$$\rho_e(t) = \frac{1}{\hbar^2}\int_{-\infty}^{\infty} e^{\mathcal{L}_e(t-t')}\rho_p(t')\,\mathrm{d}t'$$
$$= \frac{1}{\hbar^2}\int_{-\infty}^{\infty} e^{\mathcal{L}_e(t-t')}\left[|\vec{\mu}_{eg}\cdot\vec{E}_0|^2\mathcal{W}_E(t',\Delta E_{eg}^{\text{el}}/\hbar)\rho_g\right]\mathrm{d}t' \tag{53}$$

and provides a clear interpretation of the photoexcitation process. For each time t', the stationary ground-state density is multiplied by the Wigner pulse transform at time t' and by the squared projection of the transition dipole moment on the electric field amplitude $|\vec{\mu}_{eg}\cdot\vec{E}_0|^2$ – we denote this the promoted density ρ_p. The density promoted at time t' is then propagated in the excited electronic state from time t' until the desired time t. Finally, integration over all the times t' reconstructs the excited-state density at time t.

As stated earlier, Eq. (53) is formally valid only for times t after the pulse duration since we changed the upper integration limit from t to ∞ in Eq. (37). This modification was necessary to obtain the Wigner pulse transform $\mathcal{W}_E$, which requires integration from $-\infty$ to ∞, in the equations. However, this modification leads to a loss of validity during and before

the pulse. To clarify this statement, let us examine the behavior of Eq. (53) during the pulse interaction by splitting the integral into two as

$$\rho_e(t) = \frac{1}{\hbar^2} \left(\int_{-\infty}^{t} \mathrm{e}^{\mathcal{L}_e(t-t')} \rho_p(t') \, \mathrm{d}t' + \int_{t}^{\infty} \mathrm{e}^{\mathcal{L}_e(t-t')} \rho_p(t') \, \mathrm{d}t' \right) . \tag{54}$$

The first integral takes the promoted density ρ_p excited before the current time ($t' < t$) and propagates it forward in time with $\mathrm{e}^{\mathcal{L}_e(t-t')}$. Conversely, the second integral goes over excitation times larger than the current time ($t' > t$) and, therefore, takes the promoted density in the *future* and propagates it backward in time with $\mathrm{e}^{-\mathcal{L}_e|t-t'|}$ as $t - t' < 0$. In other words, the first integral accounts for the density that has been promoted to the excited state, while the second integral takes care of the density that is yet to be promoted to the excited state. Thus, if our current time t is after the pulse, the second integral is equal to zero and we can write

$$\begin{aligned} \rho_e(t) &= \frac{1}{\hbar^2} \int_{-\infty}^{t} \mathrm{e}^{\mathcal{L}_e(t-t')} \rho_p(t') \, \mathrm{d}t' \\ &= \frac{1}{\hbar^2} \int_{-\infty}^{t} \mathrm{e}^{\mathcal{L}_e(t-t')} \left[|\vec{\mu}_{eg} \cdot \vec{E}_0|^2 \mathcal{W}_E(t', \Delta E_{eg}^{\mathrm{el}}/\hbar) \rho_g \right] \mathrm{d}t' \, . \end{aligned} \tag{55}$$

If our current time t is during or before the pulse, neglecting the second integral means that we discard the *future* excited-state density and only account for the part that has been already excited. We retain only the first integral in PDA, trying to alleviate the restriction of Eq. (53) to describe only the density for times after the pulse. The excitation picture stemming from Eq. (55) is illustrated in Fig. S3, and is easy to convert into an algorithm for modeling photoexcitation processes in pump-probe excitation, see Section 4.

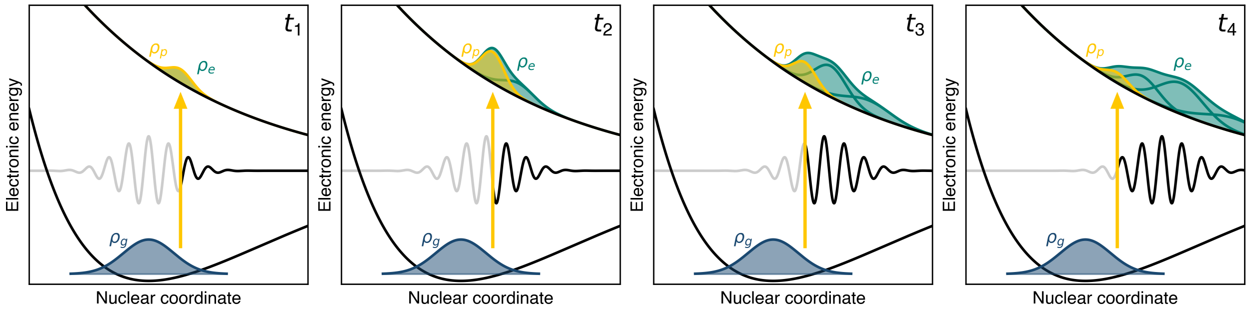

Figure S3: Illustration of the promoted density approach for photoexcitation and subsequent dynamics. The promoted density is depicted at four different times ($t_1 < t_2 < t_3 < t_4$) along with the excited-state density, which consists of propagated promoted densities from earlier times (see also Fig. S2).

The final approximation to make this scheme applicable to trajectory-based nonadiabatic dynamics approaches is to take the Wigner representation of the density operators, retaining

only the lowest order of $\hbar$. The classical excited-state density then reads

$$\boxed{\rho_e^{\text{cl}}(\underline{\mathbf{R}}, \underline{\mathbf{P}}, t) = \frac{1}{\hbar^2}\int_{-\infty}^{t} \mathrm{e}^{\mathcal{L}_e^{\text{cl}}(t-t')}\left[|\vec{\mu}_{eg}(\underline{\mathbf{R}})\cdot\vec{E}_0|^2\mathcal{W}_E(t', \Delta E_{eg}^{\text{el}}(\underline{\mathbf{R}})/\hbar)\rho_g^{\text{cl}}(\underline{\mathbf{R}}, \underline{\mathbf{P}})\right]\mathrm{d}t'\,.} \tag{56}$$

We emphasized the dependence of all the terms on nuclear positions $\underline{\mathbf{R}}$ and momenta $\underline{\mathbf{P}}$ for clarity. Eq. (56) is the final equation for simulating excited-state dynamics triggered by a laser pulse. The scheme emanating from Eq. (56) is simple: one only needs to calculate the promoted density ρ_p for all times t' during the pulse and then propagate this promoted density in the excited electronic states with the standard techniques of nonadiabatic dynamics, e.g., trajectory surface hopping or *ab initio* multiple spawning. Eq. (56) also provides a simple recipe to construct the promoted density ρ_p: take the ground-state density and multiply it by the Wigner pulse representation and the squared projection of the transition dipole moment on the electric field amplitude.

Let us finish this Section by summarizing the different assumptions and approximations made to derive Eq. (56).

1. Eq. (56) was derived within the framework of first-order perturbation theory with all its assumptions, meaning that this equation cannot be used for two-photon processes, strong-field regimes, etc.

2. The off-diagonal matrix elements, like the nonadiabatic coupling terms, were considered to be zero in our starting Hamiltonian. This assumption decouples the photoexcitation process from the nonadiabatic ones and might not be well justified if the nonadiabatic couplings are non-negligible in the Franck-Condon region.

3. PDA was initially derived to describe the nuclear density in the excited state after the laser pulse, yet we attempted to alleviate this approximation in Eq. (55), without affecting the obtained excited-state density after the pulse. This modification improves the dynamics during the laser pulse (see Figs. S7 and S10), however, it should be taken with care.

4. The derivation of Eq. (56) assumes a position-dependent transition dipole moment, utilizing the Baker–Campbell–Hausdorff formula to first order. To assess the validity of this assumption, we discuss here the importance of the second-order term. In the following, we will use the notation $\tilde{\mu} = \vec{\mu}_{eg}\cdot\vec{E}_0$ for simplicity. The second-order Baker–Campbell–Hausdorff formula reads

$$\mathrm{e}^{-\frac{i}{\hbar}\hat{H}_e\frac{s}{2}}\tilde{\mu} = \mathrm{e}^{-\frac{i}{\hbar}\hat{H}_e\frac{s}{2}}\mathrm{e}^{\ln(\tilde{\mu})} \overset{\text{BCH}}{\approx} \mathrm{e}^{-\frac{i}{\hbar}\hat{H}_e\frac{s}{2}+\ln(\tilde{\mu})-\frac{is}{4\hbar}\left[\hat{T},\ln(\tilde{\mu})\right]}\,, \tag{57}$$

where the commutator takes the form

$$\left[\hat{T}, \ln(\tilde{\mu})\right] = -\frac{i\hbar}{2m}\left(\hat{p}\frac{\mathrm{d}\ln(\tilde{\mu})}{\mathrm{d}\hat{x}} + \frac{\mathrm{d}\ln(\tilde{\mu})}{\mathrm{d}\hat{x}}\hat{p}\right) = \frac{\hbar^2}{2m}\left[\left(\frac{\tilde{\mu}'}{\tilde{\mu}}\right)^2 - \frac{\tilde{\mu}''}{\tilde{\mu}} - 2\frac{\tilde{\mu}'}{\tilde{\mu}}\frac{\mathrm{d}}{\mathrm{d}x}\right]. \tag{58}$$

Hence, the first correction to the transition dipole moment will be small if (i) the derivatives of the transition dipole moment vary slowly compared to its value and (ii) the laser pulses are short as we integrate the term over the pulse duration. The approximation might not be justified for long laser pulses and quickly varying transition dipole moments.

5. We have also used a short-time approximation by using the first-order Baker–Campbell–Hausdorff formula for the propagator $\mathrm{e}^{-\frac{i}{\hbar}\hat{H}_e\frac{s}{2}}\mathrm{e}^{\frac{i}{\hbar}\hat{H}_g\frac{s}{2}}$. This approximation can again be relaxed by considering the second-order term

$$\mathrm{e}^{-\frac{i}{\hbar}\hat{H}_e\frac{s}{2}}\mathrm{e}^{\frac{i}{\hbar}\hat{H}_g\frac{s}{2}} \overset{\mathrm{BCH}}{\approx} \mathrm{e}^{-\frac{i}{\hbar}\Delta E^{\mathrm{el}}_{eg}\frac{s}{2}-\frac{1}{8\hbar^2}\left[\hat{T},\Delta E^{\mathrm{el}}_{eg}\right]s^2}, \tag{59}$$

where the commutator can be written as

$$\left[\hat{T},\Delta E^{\mathrm{el}}_{eg}\right] = -\frac{i\hbar}{2m}\left(\hat{p}\frac{\mathrm{d}\Delta E^{\mathrm{el}}_{eg}}{\mathrm{d}\hat{x}} + \frac{\mathrm{d}\Delta E^{\mathrm{el}}_{eg}}{\mathrm{d}\hat{x}}\hat{p}\right) = -\frac{\hbar^2}{2m}\left(\frac{\mathrm{d}^2\Delta E^{\mathrm{el}}_{eg}}{\mathrm{d}x^2} + 2\frac{\mathrm{d}\Delta E^{\mathrm{el}}_{eg}}{\mathrm{d}x}\frac{\mathrm{d}}{\mathrm{d}x}\right). \tag{60}$$

Combining these two expressions together results in

$$\mathrm{e}^{-\frac{i}{\hbar}\hat{H}_e\frac{s}{2}}\mathrm{e}^{\frac{i}{\hbar}\hat{H}_g\frac{s}{2}} \overset{\mathrm{BCH}}{\approx} \mathrm{e}^{-\frac{i}{\hbar}\Delta E^{\mathrm{el}}_{eg}\frac{s}{2}+\frac{1}{16m}\left(\frac{\mathrm{d}^2\Delta E^{\mathrm{el}}_{eg}}{\mathrm{d}x^2}+2\frac{\mathrm{d}\Delta E^{\mathrm{el}}_{eg}}{\mathrm{d}x}\frac{\mathrm{d}}{\mathrm{d}x}\right)s^2}. \tag{61}$$

As stressed in point 4 above, this analysis means that Eq. (56) might not be justified for long laser pulses and significant nuclear gradient differences.

Let us try to establish a range of validity to neglect the second-order contribution. If one retains only the first term on the right-hand side of Eq. (60) (as the derivative makes the exponential difficult to evaluate), the pulse duration should follow the following condition:

$$\frac{1}{8m}\frac{\mathrm{d}^2\Delta E^{\mathrm{el}}_{eg}}{\mathrm{d}x^2}\tau^2 < 1. \tag{62}$$

Note that the factor 8 instead of 16 appears because we have the exponential twice in the derivation. Considering the photoexcitation of NaI from its ground-state minimum, we obtain $\tau < 45$ fs.

6. The transition to classical nuclear densities assumed the truncation of the terms with a higher order in $\hbar$ in the exact Wigner representation of the quantum operators. The approach is then not suitable for situations where strong quantum effects play a role (which is compatible in any case with the use of most trajectory-based approaches to nonadiabatic dynamics). As such, we expect PDA to be valid for molecular cases where the nuclear ensemble method is capable of adequately describing absorption spectra.

4 Algorithm to use the promoted density approach for single and multiple excited states

In the main text, we have proposed a practical implementation of Eq. (56), coined promoted density approach (PDA). We provide here its algorithmic description (Algorithm 1). The algorithm randomly selects the (nuclear) position-momentum pairs $\{\underline{\mathbf{R}}_i, \underline{\mathbf{P}}_i\}$ from the (approximate) ground-state density, randomly selects an excitation time t', and calculates a probability p based on Eq. (56). At that stage, negative probabilities must be handled if they occur. The probability p is then compared to a random number generated from a uniform distribution in the range 0 to p_{max}, where p_{max} is the maximum probability that can be determined from the available position-momentum pairs and excitation times.

Algorithm 1: The PDA algorithm used to sample the promoted density $\rho_p^{\text{cl}}(\underline{\mathbf{R}}, \underline{\mathbf{P}}, t')$. The input consists of the ground-state nuclear position-momentum pairs $\{\underline{\mathbf{R}}_i, \underline{\mathbf{P}}_i\}$ with their corresponding excitation energies $\Delta E_i = \Delta E_{eg}^{\text{el}}(\underline{\mathbf{R}}_i)$ and transition dipole moments $\vec{\mu}_i = \vec{\mu}_{eg}(\underline{\mathbf{R}}_i)$.

```
estimate maximum probability p_max
j = 1
while j ≤ N_p do
    randomly select i ∈ {1, ..., N_g}
    randomly select t'
    calculate probability p = |μ_i · E_0|^2 W_E(t', ΔE_i)
    if p < 0 then
        handle negative probabilities (see Section 6.4)
    end
    randomly select R ∈ [0, p_max]
    if R ≤ p then
        accept {R_i, P_i, t'} as an initial condition j
        j = j + 1
    end
end
```

The algorithm above can be easily extended for multiple excited states. Considering more excited states in the derivation proposed in Section 3 means that first-order perturbation theory would provide us with the same uncoupled formula (Eq. (36)) for each excited electronic state. Thus, we would end up with an Eq. (56) for each electronic state considered. Hence, extending PDA to multiple electronic states requires only one additional step: randomly selecting the excited state s from a set of N_s excited states, see Algorithm 2. Note that in such a case, the initial conditions generated by PDA contain additional information about the excited state in which the nonadiabatic dynamics should be initiated, i.e., $\{\underline{\mathbf{R}}_j, \underline{\mathbf{P}}_j, t'_j, s_j\}$.

Algorithm 2: The PDA algorithm used to sample the promoted densities $\rho_p^{\text{cl},s}(\underline{\mathbf{R}}, \underline{\mathbf{P}}, t')$ considering multiple excited electronic states denoted by the index s. The input consists of the ground-state nuclear position-momentum pairs $\{\underline{\mathbf{R}}_i,\ \underline{\mathbf{P}}_i\}$ with their corresponding excitation energies $\Delta E_i^s = \Delta E_{sg}^{\text{el}}(\underline{\mathbf{R}}_i)$ and transition dipole moments $\vec{\mu}_i^s = \vec{\mu}_{sg}(\underline{\mathbf{R}}_i)$ from the ground state g to one of the N_s excited electronic states considered.

```
estimate maximum probability p_max
j = 1
while j ≤ N_p do
    randomly select i ∈ {1, . . . , N_g}
    randomly select s ∈ {1, . . . , N_s}
    randomly select t'
    calculate probability p = |μ_i^s · E_0|^2 W_E(t', ΔE_i^s)
    if p < 0 then
        handle negative probabilities (see Section. 6.4)
    end
    randomly select R ∈ [0, p_max]
    if R ≤ p then
        accept {R_i, P_i, t', s} as an initial condition j
        j = j + 1
    end
end
```

5 Computational details

5.1 NaI – sodium iodide

The reference (numerically-exact) quantum dynamics simulations were performed with the split-operator technique in the diabatic basis using a time step of 0.25 a.u. and on a grid with 8192 points between 3.7 and 75 a.u. The convergence of the results with respect to the grid and the time step was thoroughly tested. The NaI diabatic Hamiltonian was reproduced from Ref. S3 (originally coming from Ref. S7) and is defined as

$$\mathbf{H}_{\mathrm{d}} = \hat{T}\mathbf{1}_2 + \begin{pmatrix} V_X & V_{XA} + V_{\mathrm{int}}(t) \\ V_{XA} + V_{\mathrm{int}}(t) & V_A \end{pmatrix} , \tag{63}$$

where $\hat{T}$ is the kinetic energy operator, V_X and V_A are the diabatic potential energy curves for the ionic state $X(^1\Sigma^+)$ and covalent state $A(0^+)$ respectively, V_{XA} is the diabatic coupling, and V_{int} is the interaction term defined as

$$V_{\mathrm{int}}(t) = -\vec{\mu}_{XA} \cdot \vec{E}_0 E(t) = -\mu_{XA} E_0 E(t) , \tag{64}$$

where μ_{XA} and E_0 are magnitudes of their respective vectors. The diabatic potential energy curves and the diabatic coupling are expressed in the following form:

$$V_X(R) = \left[A_2 + \left(\frac{B_2}{R}\right)^8\right] \exp\left(-\frac{R}{\rho}\right) - \frac{e^2}{R} - \frac{e^2(\lambda^+ + \lambda^-)}{2R^4} - \frac{C_2}{R^6} - \frac{2e^2\lambda^+\lambda^-}{R^7} + \Delta E_0 , \tag{65}$$

$$V_A(R) = A_1 \exp\left[-\beta_1(R - R_0)\right] , \tag{66}$$

$$V_{XA}(R) = A_{12} \exp\left[-\beta_{12}(R - R_x)^2\right] , \tag{67}$$

with all parameters summarized in Tab. S1. A visual representation of the Hamiltonian is provided in Fig. S4.

The electric field intensity E_0 was set to 0.001 a.u. to remain within the weak-field regime, resulting in a maximum of 0.05% population transfer for the longest pulse. The scalar electric field $E(t)$ is defined in Eq. (11) in the main text. The precise pulse frequencies used in the work are $\omega_0 = 0.15250790$, 0.14294844, and 0.13520905 a.u.

The bare diabatic Hamiltonian, that is, without the $V_{\mathrm{int}}(t)$ term, was transformed into the adiabatic representation for the FSSH dynamics through the following transformation matrix

$$\mathbf{U} = \begin{pmatrix} \cos\theta & \sin\theta \\ -\sin\theta & \cos\theta \end{pmatrix} , \tag{68}$$

where θ is the mixing angle defined as

$$\theta = \frac{1}{2} \arctan \frac{2V_{XA}}{V_{XX} - V_{AA}} . \tag{69}$$

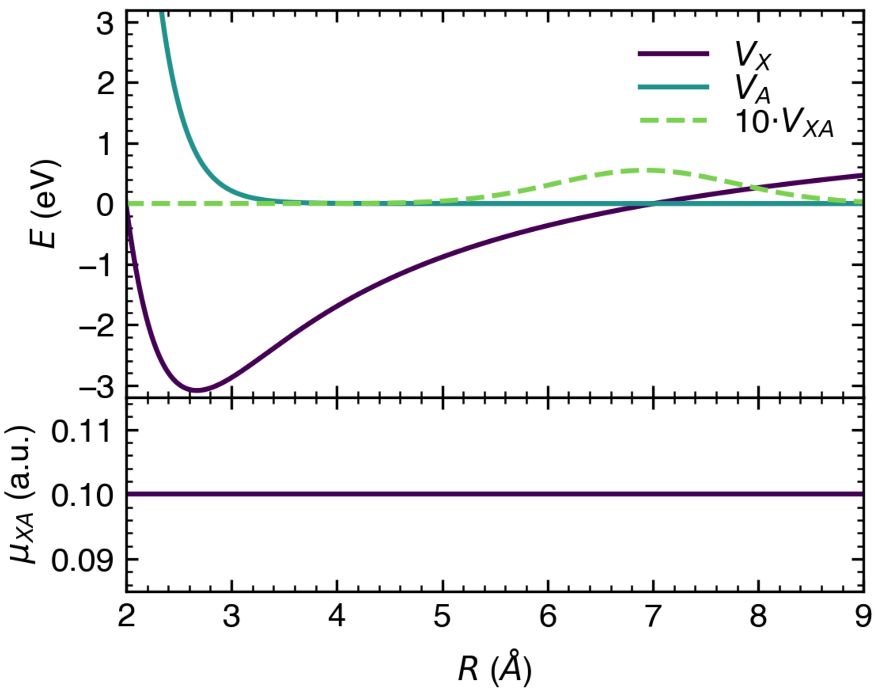

Figure S4: A visual representation of the terms used to build the NaI diabatic Hamiltonian defined in Eq. (63).

Table S1: Parameters defining the NaI diabatic Hamiltonian, reproduced from Ref. S3 (the original diabatic parameters come from Ref. S7). The mass m was taken as the reduced mass of NaI.

parameter	value
A_2	2760 eV
B_2	2.398 eV$^{1/8}$ Å
C_2	11.3 eV Å^6
λ^+	0.408 Å^3
λ^-	6.431 Å^3
ρ	0.3489 Å
ΔE_0	2.075 eV
e^2	14.3996 eV Å
A_1	0.813 eV
β_1	4.08 Å^{-1}
R_0	2.67 Å
A_{12}	0.055 eV
β_{12}	0.6931 Å^{-2}
R_x	6.93 Å
μ_{XA}	0.1 a.u.
m	35480.251398 a.u.

The adiabatic potential energy curves then read

$$E^{\mathrm{el}}_{1,2} = \frac{V_{XX} + V_{AA}}{2} \pm \frac{1}{2}\sqrt{(V_{AA} - V_{XX})^2 + 4V_{XA}^2} \tag{70}$$

and the nonadiabatic coupling vector is defined as

$$d_{12} = \langle \phi_1^{\mathrm{ad}} | \frac{d}{dR} \phi_2^{\mathrm{ad}} \rangle = -\frac{d\theta}{dR} \, . \tag{71}$$

The FSSH simulations were performed with an energy-decoherence parameter 0.1 a.u.[S8] and a time step of 2.5 a.u. using the molecular dynamics code ABIN.[S9] We note that the previous study of NaI by Martínez-Mesa and Saalfrank[S3] reported only a minor effect of the decoherence parameter on the predissociation dynamics. 10'000 position-momentum pairs were sampled from the ground-state Wigner distribution (the harmonic approximation was not invoked) and propagated with the FSSH method. We stress again here that the FSSH simulations *do not include the explicit interaction with a laser pulse*.

5.2 Protonated formaldimine

To simulate the photodynamics of protonated formaldimine, we reused 500 FSSH trajectories from our previous work on this molecule.[S10] These 145fs-long trajectories employed a time step of 0.24 fs and an energy-decoherence correction with the decoherence parameter 0.1 a.u.[S8] The electronic structure was described by the FOMO-CASCI method,[S11] considering 12 electrons in 8 orbital and a 6-31G* basis set. The Gaussian broadening parameter was set to 0.2 a.u. More details about the methodology are available in the original Ref. S10.

5.3 Calculation of observables

Calculating observables with PDA is a slightly more complex task than when the vertical sudden excitation is invoked. While all the (FSSH) trajectories are initiated in an excited state at the very same time within the sudden vertical excitation, the number of trajectories in the excited state increases gradually within PDA. This observation leads to an important question: *how should one evaluate an observable before and during the pulse when not all the trajectories have yet been promoted?*

We propose in this work to consider that each trajectory starts at time $-\infty$ and remains 'fixed' (or frozen) at their ground-state geometry until the excitation time t' is reached and the trajectory gets promoted to the excited state, initiating its evolution. This strategy is based on the Eq. (56), where the system is initially described by its stationary ground-state density ρ_g before chunks of it get promoted to the excited state. We shall illustrate this approach for the specific case of electronic-state populations. We consider the molecule in its ground state (g) until time t' when it gets promoted to the excited state. From that time, the population is governed by the nonadiabatic dynamics (starting in the excited state), in other words, by the standard population p_i^{nonad} evaluated during typical FSSH or AIMS

simulations. Hence, the time-dependent electronic state populations take the form:

$$p_i(t) = \begin{cases} g & t < t' \\ p_i^{\text{nonad}}(t) & t > t' \, . \end{cases} \tag{72}$$

Conversely, evaluating observables in a specific electronic state (s) is simply based on trajectories active in that state at any given moment, i.e.,

$$\mathcal{O}^s(t) = \frac{\sum_{i=1}^{N^s(t)} \mathcal{O}_i^s(t)}{N^s(t)} \, , \tag{73}$$

where $\mathcal{O}^s$ is the desired observable in the electronic state s, $N^s(t)$ is the time-dependent number of trajectories propagated in the state s, and $\mathcal{O}_i^s$ is the observable evaluated for the trajectories i. The sum is taken over all trajectories propagating in the given state s at time t.

5.3.1 Population transfer in QD and PDA

While the comparison of $\langle R \rangle_{S_1}$ and $\langle \Delta R \rangle_{S_1}$ between QD and FSSH+PDA(W) can be done directly from the simulation data due to the presence of a normalization in the formula for an expectation value, the comparison of electronic populations shown in this Supporting Information requires a normalization of the QD populations. As we mentioned at the beginning of this Section, the field intensity E_0 was set to trigger a maximum of 0.05% population transfer to the excited electronic state, ensuring that our simulations are in the weak-field limit. Contrarily, PDA considers only the promoted part of the density (0.05%) and ignores the part remaining in the ground state. Thus, the QD electronic state populations must be normalized such that they reflect only the excited part of the density. In practice, we have taken the maximum of the QD excited-state population (0.05%) and rescaled it to 1. Note that this rescaling strategy was only possible as the depopulation of the excited electronic state due to nonadiabatic transitions happens long after the pulse.

6 Extended tests of PDA and PDAW

In this Section, we present a series of extended tests of PDA and PDAW (as mentioned above and in the main article) based on the photodynamics of NaI.

6.1 Comparing PDA, PDAW, and standard windowing

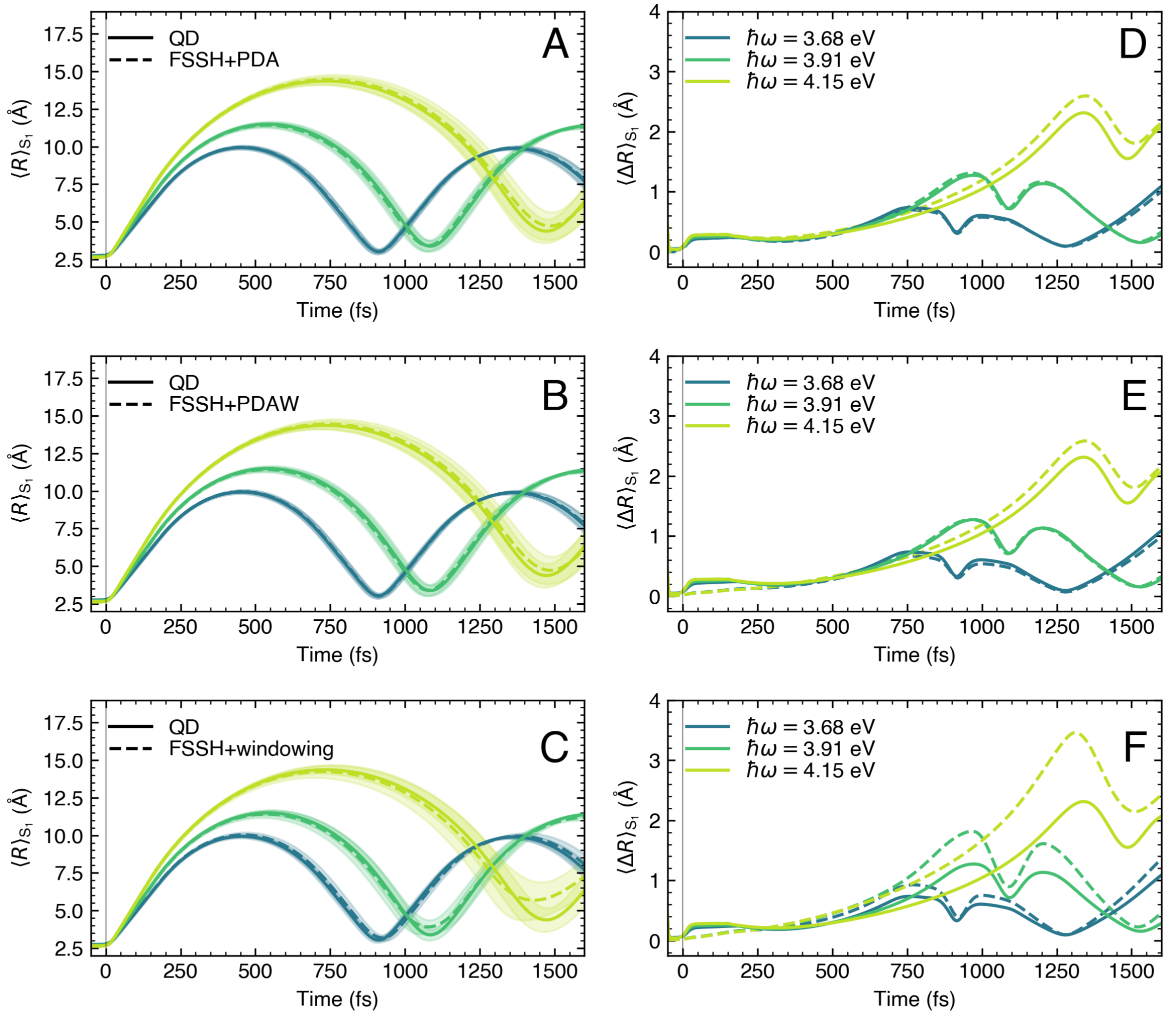

Figure S5: Supplement to Fig. 3 from the main article, including a comparison of FSSH simulations using PDAW. The results highlight the improved performance of the windowing strategy (PDAW) with respect to the most commonly used windowing approach. FSSH+PDAW matches the results of FSSH+PDA. (A) Expectation values of the NaI bond length in S_1 for three different laser pulse frequencies, comparing quantum dynamics with an explicit 20-fs laser pulse (solid lines) and PDA combined with FSSH nonadiabatic dynamics (dashed lines). The shaded area represents $\langle \Delta R \rangle_{S_1}$ of the nuclear wavepacket (QD) or trajectories (FSSH). (B) Same as in panel A but for FSSH combined with PDAW (dashed lines). (C) Same as in panel A but for FSSH using a simple windowing approach combined with a time convolution (dashed lines). (D) The width of the excited-state nuclear wavepacket $\langle \Delta R \rangle_{S_1}$ corresponding to simulations in panel A (similar correspondence for panel E and panel F).

6.2 Excitation with a chirped pulse: PDA vs. PDAW

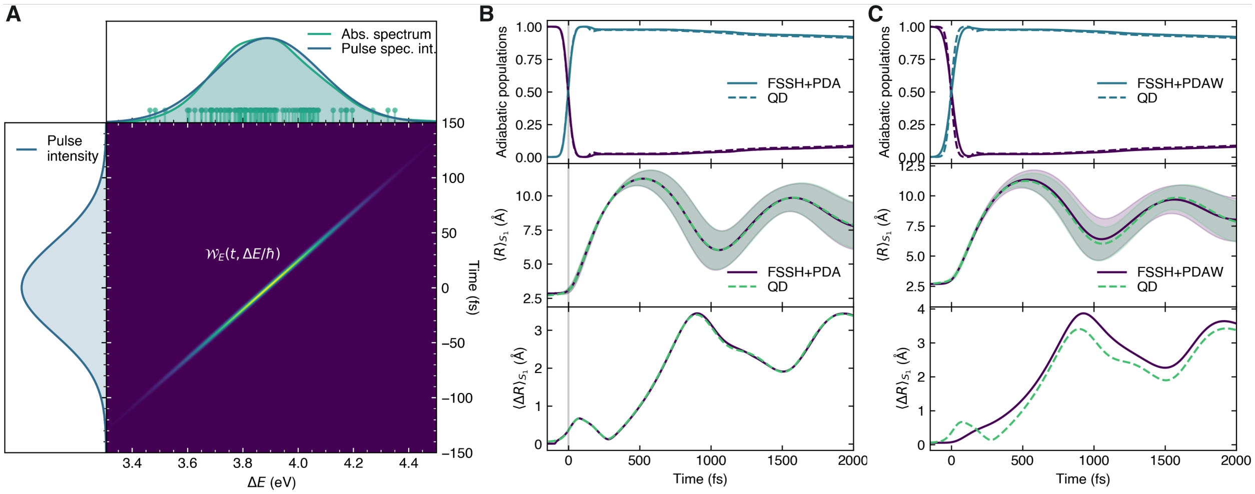

Figure S6: Photodynamics of NaI following excitation with a 100-fs Gaussian pulse with $\omega_0 = 0.14294844$ a.u. and a linear chirp parameter $\beta = 2 \times 10^{-6}$ a.u. (A) The Wigner pulse representation of the chirped Gaussian pulse plotted with the absorption spectrum, pulse intensity, and spectral intensity. (B) Adiabatic electronic state populations, expectation values of the NaI bond length in S_1, and its standard deviation $\langle \Delta R \rangle_{S_1}$ for PDA combined with FSSH nonadiabatic dynamics (dashed lines), compared to the reference QD with an explicit laser pulse (solid lines). (C) Same as in panel B but for PDAW. FSSH+PDA is in excellent agreement with the QD results and outperforms FSSH+PDAW. Although not quantitative, FSSH+PDAW can still capture the pulse effects qualitatively, demonstrating a weak effect of the chirp.

6.3 Long laser pulses with PDA

As discussed in Section 3, one of the approximations behind PDA assumes a short duration of the laser pulse. Based on the second-order Baker–Campbell–Hausdorff (BCH) formula, we have estimated (in the specific case of the photodynamics of NaI) that the pulse duration should be shorter than 45 fs for this approximation to be valid. So far, we have applied only pulses with a maximum value of $\tau = 20$ fs, well below this estimated limit. Hence, we present here additional simulations with 100-fs and 500-fs laser pulses to explore the boundaries of the BCH approximation. The results presented in Fig. S7 reveal that the effect of the 100-fs laser pulse is still perfectly captured by PDA. For the 500-fs pulse, we observe a sizeable deviation from the QD result. However, we would rather attribute this deviation to quantum interferences within the excited nuclear wavepacket caused by the long laser pulse, since the period of oscillation in the excited state is similar to the pulse duration. Still, FSSH+PDA manages to depict the adiabatic nuclear dynamics qualitatively, while the time evolution of the adiabatic populations and the nuclear wavepacket width are better captured. These results indicate that PDA is more robust with respect to the laser pulse duration than one would expect from our estimated limit.

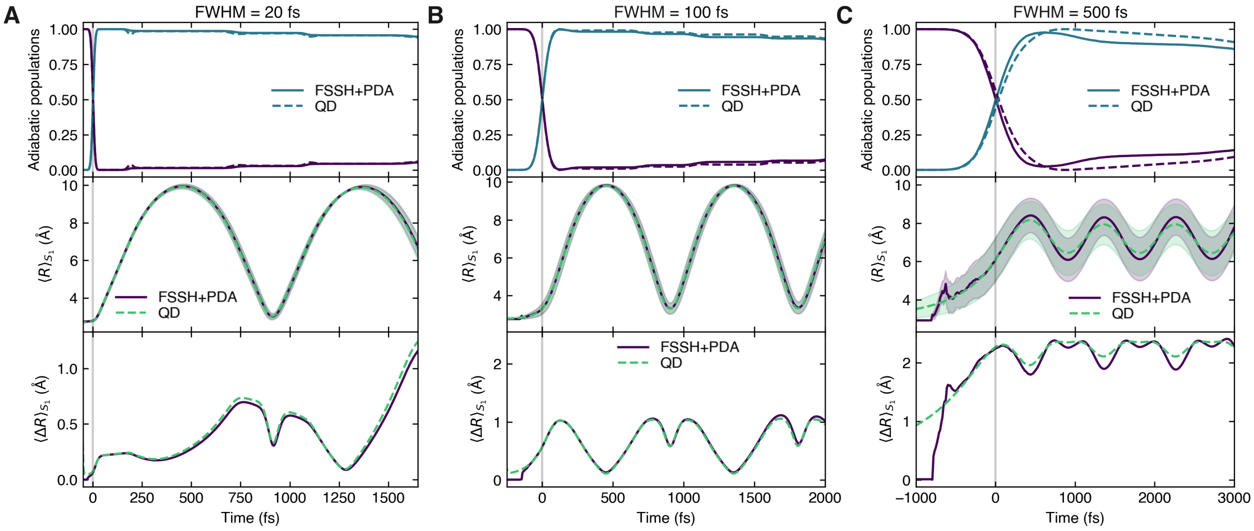

Figure S7: Photoexcitation of NaI triggered by a Gaussian laser pulse with a frequency $\omega_0 = 0.13520905$ a.u. and three different FWHM parameter τ, comparing QD to FSSH combined with PDA. (A) Adiabatic electronic state populations, expectation values of the NaI bond length in S_1, and its standard deviation $\langle \Delta R \rangle_{S_1}$ for a 20-fs laser pulse. PDA combined with FSSH nonadiabatic dynamics (dashed lines) is compared to the reference QD with an explicit pulse (solid lines). (B) Same as in panel A but for a 100-fs laser pulse. (C) Same as in panel A but for a 500-fs laser pulse.

6.4 Lorentzian envelope: negative Wigner probabilities and dynamics during the pulse

The use of a Gaussian laser pulse in the main text and previous Sections is an ideal case for PDA, since the Wigner representation of Gaussian pulses is strictly positive. However, we show in the following that PDA can also be combined with other pulse envelopes once a suitable strategy for dealing with negative probabilities stemming from $\mathcal{W}_E$ is introduced. Although $\mathcal{W}_E$ should not be seen as a probability function, the PDA scheme considers $\mathcal{W}_E$ to be a probability, leading to issues when negative values appear (for non-Gaussian laser pulses). First, let us define the Lorentzian envelope,

$$\varepsilon(t) = \left[1 + \frac{4}{1+\sqrt{2}}\left(\frac{t}{\tau}\right)^2\right]^{-1}, \tag{74}$$

where τ is the FWHM parameter for the intensity $I(t) \approx \varepsilon^2(t)$. The Wigner pulse representation of the Lorentzian envelope is shown in Fig. S8, highlighting its negative regions. The Gaussian envelope is also provided for comparison. Notice that the negative values emerge at the 'outskirts' of $\mathcal{W}_E$, with small magnitudes in comparison to the maximum of the function. As such, the negative regions appear as a minor contribution to $\mathcal{W}_E$ and it could be tempting to neglect them.

Based on this analysis, we propose two schemes for creating a modified Wigner pulse representation $\tilde{\mathcal{W}}_E$ that can handle the negative values of $\mathcal{W}_E$. First, one can consider the absolute value of $\mathcal{W}_E$, i.e., $\tilde{\mathcal{W}}_E = |\mathcal{W}_E|$. In this case, the negative regions contribute to $\mathcal{W}_E$ with small positive values, and the ground-state density can also be promoted to the excited state in these regions of t and ω. The second strategy consists of ignoring the negative values completely, i.e., substituting the negative values with zeros:

$$\tilde{\mathcal{W}}_E = \begin{cases} 0 & \text{if } \mathcal{W}_E(t,\omega) < 0 \\ \mathcal{W}_E(t,\omega) & \text{elsewhere} \end{cases} \tag{75}$$

One could also consider PDAW as a strategy to handle negative values, since $I(t)$ and $S(\omega)$ are both positive functions. We tested all the aforementioned strategies on the photoexcitation of NaI triggered by a Lorentzian pulse with $\omega_0 = 0.13520905$ a.u. and $\tau = 20$ fs, see Fig. S9. The results in Fig. S9 clearly demonstrate the ability of all these strategies to handle negative probabilities and capture the laser pulse effects at an almost quantitative level. The deviation from QD, observed for the populations and $\langle R \rangle_{S_1}$, are nearly negligible. The only significant difference can be seen for the nuclear wavepacket width $\langle \Delta R \rangle_{S_1}$, but it is still minor when compared to simulations using the sudden vertical excitation. Thus, all strategies to deal with negative values in the Wigner pulse representation appear to work for this test system, and we would favor neglecting the negative values based on the slightly better agreement in the nuclear wavepacket width between FSSH+PDA and the QD reference.

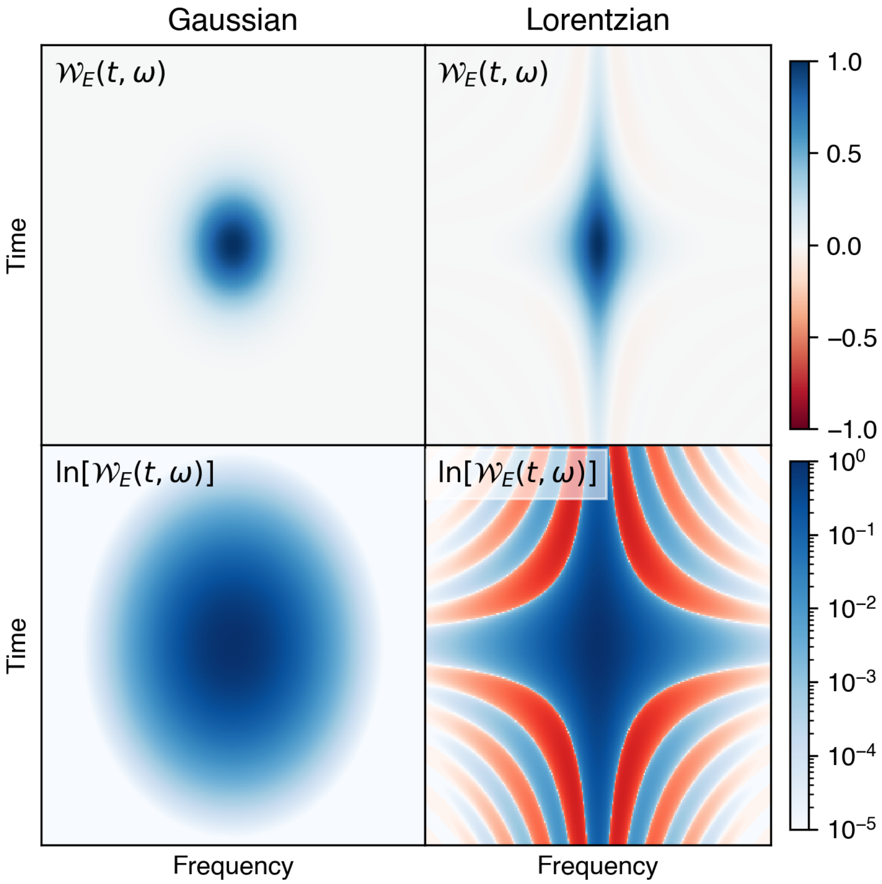

Figure S8: The Wigner representation of a Gaussian and a Lorentzian laser pulse. Blue depicts positive values of the corresponding function, while red highlights its negative parts. The top panels show $\mathcal{W}_E$ using a linear scale, demonstrating the small contribution of negative values to the overall $\mathcal{W}_E$. The bottom panels show $\mathcal{W}_E$ in a logarithmic scale, which emphasizes the structure of negative regions (barely visible using a linear standard scale).

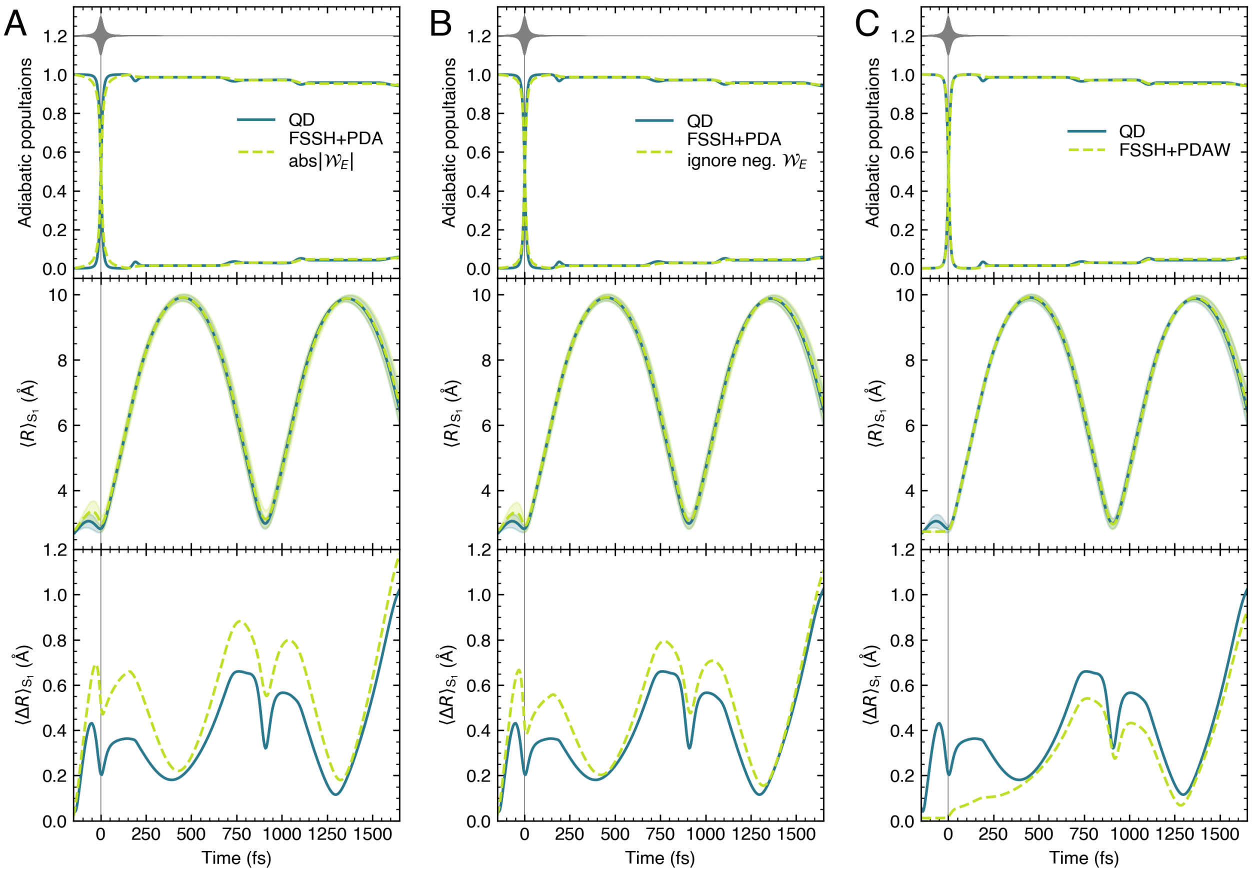

Figure S9: Photoexcitation of NaI with a Lorentzian laser pulse using a frequency $\omega_0 = 0.13520905$ a.u. and a FWHM parameter $\tau = 20$ fs. The results of QD with an explicit laser pulse are compared to those of FSSH with either PDA or PDAW. (A) Comparing QD (solid lines) with FSSH combined with PDA (dashed lines), taking the absolute value of $\mathcal{W}_E$. The adiabatic populations, expectation values of the NaI bond length in S_1, and its standard deviation $\langle \Delta R \rangle_{S_1}$ are reported. (B) Same as in panel A, but this time simply ignoring the negative values of $\mathcal{W}_E$, i.e., setting them to 0. (C) Same as in panel A, but using PDAW instead of PDA.

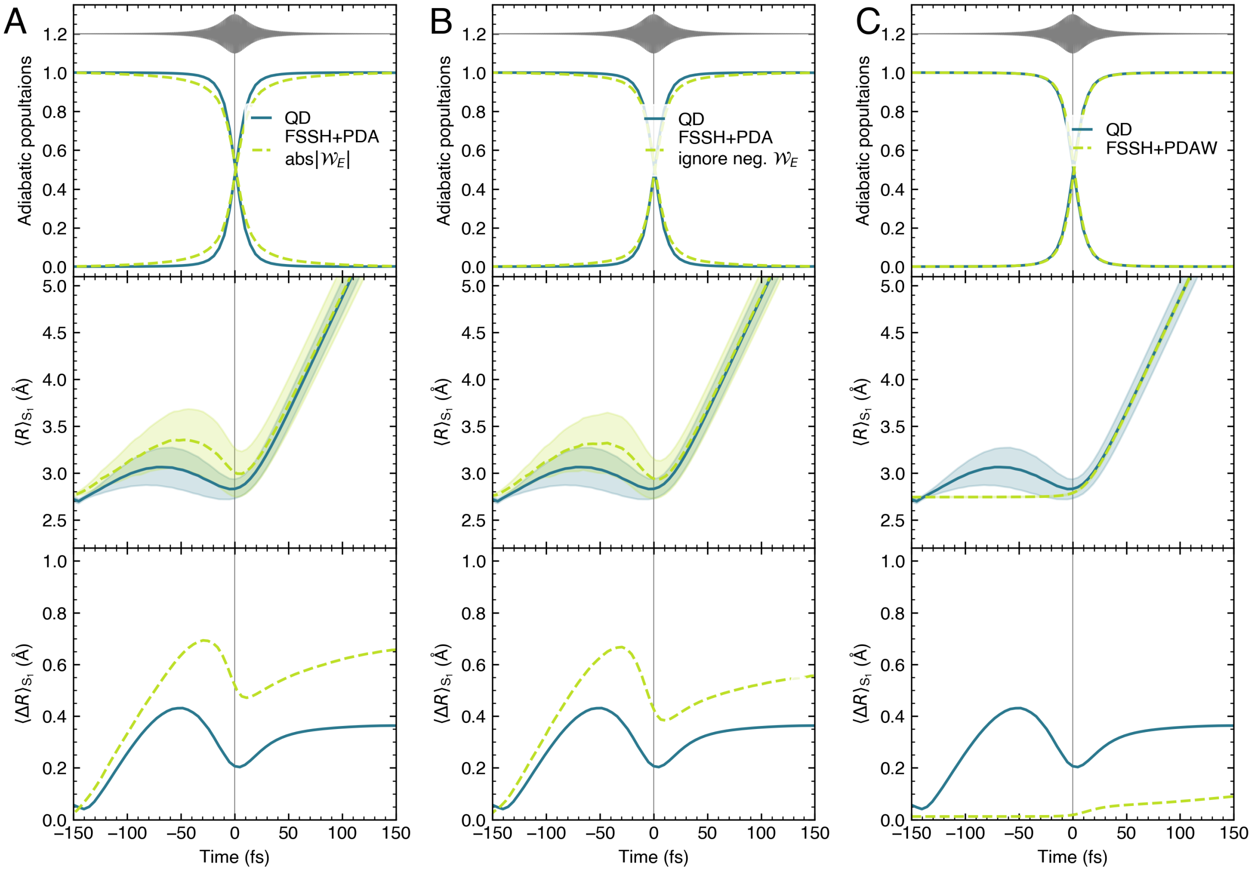

Figure S10: Zoom of the data reported in Fig. S9 in the time window around the laser pulse (from -150 fs to 150 fs). The data demonstrate the ability of PDA to account for the nuclear dynamics also during the laser pulse (missed, as expected, by PDAW).

7 Python implementation of PDA and PDAW

Both PDA and PDAW were implemented in a user-friendly Python code called `promdens`,[S12] available to the public on GitHub or as a Python package via PyPI. The code generates the initial conditions (PDA) or weights and convolution parameters (PDAW) that can be used to run and process trajectory-based simulations from any nonadiabatic dynamics code. In this Section, we provide a brief set of guidelines on how to use this code, and we direct the interested reader to the GitHub repository for more details and the manual.

7.1 Installation

The code is published on PyPI and can be installed with `pip`

```
$ pip install promdens
```

After installation, the code is available as a script via the `promdens` command. To print help, run:

```
$ promdens --help
```

The minimum supported Python version is 3.7. The code depends on `numpy` and `matplotlib` libraries that are automatically installed by `pip`.

7.2 Usage

The code requires information about the method (PDA or PDAW), the number of excited states, the number of initials conditions to be generated, and the characteristics of the laser pulse, such as the envelope type (Gaussian, Lorentzian, sech, etc.), the pulse frequency, the linear chirp parameter, and the full width at half maximum parameter. The code can be launched from a terminal with a series of flags as follows

```
$ promdens --method pda --energy_unit a.u. --tdm_unit debye --nstates 2 ↩
   --fwhm 3 --omega 0.355 --npsamples 10 --envelope_type gauss ↩
   input_file.dat
```

The input file should contain information about the excitation energies and magnitudes of the transition dipole moments for each pair of sampled nuclear positions and momenta (label by an index number).[c] In the following, we provide an example of the input file for the first two excited states of protonated formaldimine:

```
#index     dE12 (a.u.)  |mu_12| (Debye)  dE13 (a.u.)  |mu_13| (Debye)
1          0.32479719       0.1251       0.40293672       1.351
2          0.32070472       0.2434       0.40915241       1.289
3          0.34574925       0.7532       0.38595754       1.209
4          0.33093699       0.1574       0.36679075       1.403
```

[c] If the user would like to consider the pulse polarization $\vec{E}_0$ as well, the quantity $|\vec{\mu}_{eg} \cdot \vec{E}_0|$ should then be provided.

```
5          0.31860215        0.1414        0.36973886        1.377
6          0.31057768        0.0963        0.40031651        1.390
7          0.33431888        0.1511        0.40055704        1.358
8          0.31621589        0.0741        0.36644659        1.425
9          0.32905912        0.5865        0.36662982        1.277
10         0.31505412        0.2268        0.35529522        1.411
```

Using this input file and running the command line above, the user receives the following output file called `pda.dat` containing information about excitation times and initial excited states:

```
# Sampling: number of ICs = 10, number of unique ICs = 5
# Field parameters: omega = 3.55000e-01 a.u., linear_chirp = 0.00000e+00
    a.u., fwhm = 3.000 fs, t0 = 0.000 fs, envelope type = 'gauss'
# index  exc. time (a.u.)  el. state     dE (a.u.)    |tdm| (a.u.)
     3     15.09731061         1        0.34574925     0.29635106
     3     25.94554064         1        0.34574925     0.29635106
     3     61.98106992         1        0.34574925     0.29635106
     4      7.38522206         2        0.36679075     0.55201877
     8    -14.27561557         2        0.36644659     0.56067480
     9    155.72500917         2        0.36662982     0.50244331
     9    -44.31379959         2        0.36662982     0.50244331
    10     94.19109952         2        0.35529522     0.55516642
    10     -9.13220842         2        0.35529522     0.55516642
    10     31.75086044         2        0.35529522     0.55516642
```

Inspecting this output file shows that the code generated 10 initial conditions accounting for the effect of the laser pulse, yet only 5 unique ground-state samples (pairs of nuclear positions and momenta) were used: indexes 3, 9, and 10 were selected more than once. The initial conditions are also spread over both excited states. The user should then run only 5 nonadiabatic simulations: initiating the nuclear position-momentum pair with index 3 in the first excited state and the nuclear position-momentum pairs with indexes 4, 8, 9, and 10 in the second excited state.

If the same command were to be used with PDAW instead of PDA (`--method pdaw`), the output file would look as follows

```
# Convolution: I(t) = exp(-4*ln(2)*(t-t0)^2/fwhm^2)
# Parameters:  fwhm = 3.000 fs, t0 = 0.000 fs
# index         weight S1         weight S2
       1       1.78475e-05       9.66345e-07
       2       1.56842e-05       2.59858e-08
       3       6.31027e-02       1.29205e-03
       4       1.79107e-04       1.62817e-01
       5       2.31817e-06       1.01665e-01
       6       2.96548e-08       3.90152e-06
       7       3.81650e-04       3.33694e-06
```

```
       8      2.36147e-07      1.75628e-01
       9      1.47188e-03      1.37747e-01
      10      1.33347e-06      3.55670e-01
```

The code provides the pulse intensity and weights necessary for the convolution described in Eq. (15) in the main text. Note that the intensity should be normalized before being used in the convolution. If only a restricted number of trajectories can be calculated, the user should choose the indexes and initial excited states corresponding to the largest weights in the file. For example, if one could run only 10 trajectories for the protonated formaldimine, we would run the nuclear position-momentum pairs with indexes 3, 4, 7, and 9 starting in S_1 and indexes 3, 4, 5, 8, 9, and 10 starting in S_2.

If the user selects the option `--plot`, the code will produce a series of plots analyzing the provided data and calculated results, e.g. the absorption spectrum calculated with the nuclear ensemble approach, the pulse spectrum, or the Wigner pulse transform.

More information about the code is available in the manual on the GitHub repository (the code is also thoroughly commented).

References

(S1) Diels, J.-C.; Rudolph, W. In *Ultrashort Laser Pulse Phenomena (Second Edition)*, second edition ed.; Diels, J.-C., Rudolph, W., Eds.; Academic Press: Burlington, 2006; pp 1–60.

(S2) Madsen, L. B. Gauge invariance in the interaction between atoms and few-cycle laser pulses. *Physical Review A* **2002**, *65*, 053417.

(S3) Martínez-Mesa, A.; Saalfrank, P. Semiclassical modelling of finite-pulse effects on non-adiabatic photodynamics via initial condition filtering: The predissociation of NaI as a test case. *Journal of Chemical Physics* **2015**, *142*, 194107.

(S4) Li, Z.; Fang, J.-Y.; Martens, C. C. Simulation of ultrafast dynamics and pump–probe spectroscopy using classical trajectories. *Journal of Chemical Physics* **1996**, *104*, 6919–6929.

(S5) Shen, Y. C.; Cina, J. A. What can short-pulse pump-probe spectroscopy tell us about Franck-Condon dynamics? *Journal of Chemical Physics* **1999**, *110*, 9793–9806.

(S6) Meier, C.; Engel, V. Time-resolved photoelectron spectroscopy of molecular dissociation: Classical trajectory versus quantum wave-packet calculations. *Physical Chemistry Chemical Physics* **2002**, *4*, 5014–5019.

(S7) Engel, V.; Metiu, H. A quantum mechanical study of predissociation dynamics of NaI excited by a femtosecond laser pulse. *Journal of Chemical Physics* **1989**, *90*, 6116–6128.

(S8) Granucci, G.; Persico, M. Critical appraisal of the fewest switches algorithm for surface hopping. *Journal of Chemical Physics* **2007**, *126*.

(S9) Hollas, D.; Suchan, J.; Ončák, M.; Slavíček, P. PHOTOX/ABIN: Pre-release of version 1.1, https://github.com/ PHOTOX/ABIN. 2019; https://doi.org/10.5281/zenodo.1228463.

(S10) Suchan, J.; Janoš, J.; Slavíček, P. Pragmatic Approach to Photodynamics: Mixed Landau–Zener Surface Hopping with Intersystem Crossing. *Journal of Chemical Theory and Computation* **2020**, *16*, 5809–5820.

(S11) Slavíček, P.; Martínez, T. J. Ab initio floating occupation molecular orbital-complete active space configuration interaction: An efficient approximation to CASSCF. *Journal of Chemical Physics* **2010**, *132*, 234102.

(S12) Janoš, J.; Hollas, D. PROMDENS: Promoted Density Approach code. 2024; https://doi.org/10.5281/zenodo.13853643, Accessed: 2024-09-28.
