# Supplementary material for: Including Photoexcitation Explicitly in Trajectory-Based Nonadiabatic Dynamics at No Cost
Source: J Phys Chem Lett. 2024 Oct 15;15(42):10614–22. doi: 10.1021/acs.jpclett.4c02549 (PMC11514012; doi:10.1021/acs.jpclett.4c02549)
Supplement: Supplementary file 2 — jz4c02549_si_002.pdf [file jz4c02549_si_002.pdf]

jz-2024-02549s.R1

Name: Peer Review Information for "Including photoexcitation explicitly in trajectory-based nonadiabatic dynamics at no cost"

FirstRound of Reviewer Comments

Reviewer: 1

Comments to the Author

This is a highly original and significant paper that provides a practical yet rigorously-based way of initializing trajectories on excited state surfaces for simulations of non-adiabatic dynamics when triggered by a laser pulse. In almost all calculations today the effect of the pulse shape, duration, intensity are hardly considered in photo-excited dynamics simulations; instead the system is simply started with the ground-state (or thermal) density elevated as is to an excited state or small group of excited states that are assumed to be populated by the laser pulse. Instead, in this paper, a practical prescription is derived, using first-order perturbation theory to excite an initial ground-state density accounting for the effect of the difference in excitation times and strengths in different parts of the nuclear density. The results and comparison with the traditional approach are convincing, and clearly-explained, and the Supplementary Material is particularly highly-polished, with very clear and carefully presented derivations, as well as explanations of limitations and approximations assumed. I recommend the article for publication in JPCLetters after the authors address the following points.

(1) The paper focusses, in a sense, on the nuclear side of the problem, and provides a prescription for the initial conditions of the nuclear trajectories that would then propagate under some trajectory-based non-adiabatic dynamics method. If I understood correctly, each trajectory is associated with a single electronic surface (need not be the same for every trajectory) that defines then the initial electronic coefficient associated with that trajectory. But physically, the laser pulse may cause an excitation to a coherent superposition of electronic states. Methods like surface-hopping tend to be used with one electronic state nevertheless occupied, that corresponding to the active state, but other methods like Ehrenfest, or the AIMS that the authors mention, could begin with each trajectory associated to several different electronic states in a coherent way. Having a coherent electronic state or a mixed state can affect the subsequent dynamics. Some thoughts on whether and how the authors' approach can be generalized to this situation would be helpful to include in the paper.

(2) Can the authors clarify in the paragraph before the first numerical demonstration of the PDA performance, if the non-adiabatic dynamics starts at time t'_j for each trajectory j even while the pulse is on? So the non-adiabatic part of the dynamics begins at different times for different trajectories? Or is it that the adiabatic evolution is used for all trajectories, whether excited or not yet, until the pulse is considered off?

(3) Regarding the numerical demonstrations: The authors plot in Fig 3 the first moment of the density in the excited state and its variance. To isolate error from the FSSH non-adiabatic method itself from the initialization procedure, should the density somehow effectively be normalized? I mean, the FSSH may predict a different transition probability to the ground-state than the exact quantum dynamics, effectively, and that would be fault of FSSH, not the fault of the authors' new initialization procedure. Likewise in Fig 4. A comment here would be useful.

Reviewer: 2

Comments to the Author

It is a nice paper, well written and presenting interesting and timely development. Indeed, taking into account pulse shape is important problem. My only suggestion is to reference the paper "Floquet Hamiltonian for incorporating electronic excitation by a laser pulse into simulations of non-adiabatic dynamics" in Chemical Physics Volume 515, Pages 46-51, where another approach to taking into account excitation during the pulse shape has been suggested. In that paper it was demonstrated that excitation during the pulse can be significant and a method to take it into account has been suggested. The difference is that Floquet approach treats field quantum mechanically, not as a classical perturbation employed in this work. I believe the alternative Floquet technique would be worth mentioning here.

Reviewer: 3

Comments to the Author

The manuscript "Including photoexcitation explicitly in trajectory-based nonadiabatic dynamics at no cost" by Janǒs, Slavíček and Curchod discusses the importance of including explicitly the effect of the photoexcitation in the initialization of a nonadiabatic process and develops a numerical strategy to do it in an efficient way for trajectory-based methods. The theory is developed succinctly but clearly in the main text and is supported by convincing numerical results describing the photodissociation of NaI and the photoisomerization of protonated formaldimine. In addition, the

Supporting Information (SI) presents extensively the theoretical framework, includes computational details, and describes the algorithm.

The major advance reported in this work is the *strategy* proposed by the authors. This strategy aims to include efficiently and accurately the effect of the excitation via a laser pulse in a trajectory-based simulation of excited-state dynamics. Such strategy is based on a rigorous quantum-mechanical description of the excitation processes treated within perturbation theory and allows the authors to derive an algorithm that is readily applicable. In addition, the importance of treating explicitly the excitation is demonstrated in two cases, namely for NaI and protonated formaldimine.

The immediate significance of this advance is the possibility of running simulations of nonadiabatic dynamics that provide and direct comparison with experiments of photochemistry, since they are able to describe quite realistically the experimental excitation process. In addition, the strategy proposed by the authors can be even used as a post-processing tool, for instance allowing to reuse existing trajectories without the need to rerun expensive nonadiabatic simulations.

My overall assessment of the manuscript is that it is an excellent work. Therefore, I can recommend publication in The Journal of Physical Chemistry Letters, provided that the authors address my comments below.

1) When it comes to the calculations of experimental signals, like the time-resolved electron diffraction signal mentioned by the authors in the Introduction, I think that it is common practice to convolute the numerical results with a function accounting for the final duration of the pulse. Would the strategy developed in this work have a different effect on such a signal? More generally, can the authors clarify what the main differences can be expected to be between these two procedures to account for the effect of the pulse on observables?
2) The derivation of the promoted density is very elegant and convincing, at least as long as the quantum-mechanical case is discussed: the promoted density is a “modified” ground-state density, ie, modified by the squared of the interaction with the field and by the Wigner representation of the pulse.
   a) Can the instantaneous-excitation limit be recovered from this expression?
   b) To translate the concept of promoted density from the quantum-mechanical formulation to the classical formulation is terms of trajectories, it seems to me that some sort of reweighting procedure is developed. Specifically, the ground-state initial conditions sampled without the field are promoted according to some quantity/probability, namely the squared of the interaction with the field and by the Wigner representation of the pulse. Les me consider for the moment only the simple case of a non-negative Wigner representation. Is this quantity, called *p* in the manuscript, a probability? Is it defined between 0 and 1? Also, the promoted probability density depends on the nuclear positions and on time (the time at which the excitation of a particular trajectory takes place): is this actually a probability density in the “extended” configuration-time space? While the actual numerical procedure introduced by the authors, that randomly select an initial condition and an initial time to perform the

photoexcitation, seems effective, I have difficulties in justify it in light of the quantum-mechanical equations.

c) When moving from the full quantum treatment to the semiclassical treatment that introduces the phase-space representation in Eq.(7), do the authors actually do the Wigner transform of Eq.(3) or do they replace every operator/function in Eq.(3) with its phase-space representation? I guess that the former would yield a more complicated expression than Eq.(7), and in any case this should be clarified in the text.

3) The promoted density approach (PDA) is applicable efficiently as a post-processing tool because it is based on the assumption that the photoexcitation process and the dynamics in the excited state can be disentangled. However, I would expect that some trajectories are transferred back to the ground state and perhaps have an effect on the overall nonadiabatic dynamics. Can this effect appear in the formalism developed in the manuscript?

4) The PDA is reminiscent of the approach developed by Barbatti in 10.1021/acs.jctc.0c00501, even if that was developed to account for a photoexcitation by incoherent light rather than a laser pulse. Can the authors comment on the formal and algorithmic analogies/differences of the two approaches?

Technical and minor questions:

5) In Eq.(1) and related expressions (also in the SI), V_{int} appears as an operator on the left-hand side but the symbol of operator "^" disappears on the right-hand side, I guess because it is only multiplicative in nuclear space. Perhaps this choice of convention should be defined somewhere in the text.

6) At the end of page 4, the text says that ρ_g is stationary: under which Hamiltonian? I guess $\hat{H}_g$.

7) I am not convinced that Eq.(28) in the SI is simply the nuclear wavefunction, because it is written in the electronic basis, so in my opinion this is a representation of the molecular state.

8) In the derivation described in Section 3 of the SI, I think that sometimes the authors use 0 as initial time and sometimes t_0 (see for instance Eq.(34)).

9) To derive Eq.(49) in the SI, the first step is to exponentiate the logarithm of the product of the transition dipole moment and of the field, which a function with many divergences, ie, every time the cosine is zero. Can the authors propose another derivation that does not imply introducing divergences in an initially well-behaving expression?

10) Typo: In the SI just before Eq.(26), I think that "vector field" should be replaced by "vector potential".

Author's Response to Peer Review Comments:

Basile F. E. Curchod
Professor of Theoretical Chemistry
Centre for Computational Chemistry
School of Chemistry, University of Bristol
Bristol BS8 1TS
United Kingdom
E: basile.curchod@bristol.ac.uk
T: (+44) 757 704 04 24
W: www.in-silico-photochem.com

October 7, 2024

Senior Editor
The Journal of Physical Chemistry Letters

Dear Editor,

We thank the three Reviewers for their very positive and supportive comments on our manuscript 'Including photoexcitation explicitly in trajectory-based nonadiabatic dynamics at no cost' (ID: jz-2024-02549s). We have revised the manuscript to address all the Reviewers' suggestions. We detail these changes and include responses to the Reviewer's questions below. We reproduce below the entire Reviewer comments in red for clarity, and additions/corrections to the text are denoted in blue. We also uploaded an annotated version of the revised manuscript, where the modifications are indicated.

Report of Reviewer #1

This is a highly original and significant paper that provides a practical yet rigorously-based way of initializing trajectories on excited state surfaces for simulations of non-adiabatic dynamics when triggered by a laser pulse. In almost all calculations today the effect of the pulse shape, duration, intensity are hardly considered in photo-excited dynamics simulations; instead the system is simply started with the ground-state (or thermal) density elevated as is to an excited state or small group of excited states that are assumed to be populated by the laser pulse. Instead, in this paper, a practical prescription is derived, using first-order perturbation theory to excite an initial ground-state density accounting for the effect of the difference in excitation times and strengths in different parts of the nuclear density. The results and comparison with the traditional approach are convincing, and clearly-explained, and the Supplementary Material is particularly highly-polished, with very clear and carefully presented derivations, as well as explanations of limitations and approximations assumed. I recommend the article for publication in JPCLetters after the authors address the following points.

We thank the Reviewer for their very positive comment on our manuscript.

1. The paper focusses, in a sense, on the nuclear side of the problem, and provides a prescription for the initial conditions of the nuclear trajectories that would then propagate under some trajectory-based non-adiabatic dynamics method. If I understood correctly, each trajectory is associated with a single electronic surface (need not be the same for every trajectory) that defines then the initial electronic coefficient associated with that trajectory. But physically, the laser pulse may cause an excitation to a coherent superposition of electronic states. Methods like surface-hopping tend to be used with one electronic state nevertheless occupied, that corresponding to the active state, but other methods like Ehrenfest, or the AIMS that the authors mention, could begin with each trajectory associated to several different electronic states in a coherent way. Having a coherent electronic state or a mixed state can affect the subsequent dynamics. Some thoughts on whether and how the authors' approach can be generalized to this situation would be helpful to include in the paper.

   The Reviewer has correctly identified that the PDA implementation in the current form is not suitable for excitations triggering coherent superposition of electronic states. While the

current paper has focused on excitations to a single electronic state, the approach is readily extensible to more excited states (as described in the SI). Thus, for a given excitation time, one could obtain promoted densities for all excited states and initiate the dynamics in a superposition of these states with coefficients corresponding to the square root of the density. However, this approach would lack the phase of the coefficient and would account only for the separation of the wavepacket between different electronic states. We consider the excitation to a coherent superposition as an interesting case and we plan to further explore this area in a follow-up work. A comment was added to the conclusion paragraph.

> Page 16: The technique is easy to extend to multiple electronic states yet further development is needed to describe the coherent superposition of electronic states within the present framework.

2. Can the authors clarify in the paragraph before the first numerical demonstration of the PDA performance, if the non-adiabatic dynamics starts at time t'_j for each trajectory j even while the pulse is on? So the non-adiabatic part of the dynamics begins at different times for different trajectories? Or is it that the adiabatic evolution is used for all trajectories, whether excited or not yet, until the pulse is considered off?

   The nonadiabatic dynamics begins for each trajectory j at its corresponding excitation time t'_j, which is different for each trajectory. This time corresponds to the time when the molecule is promoted from the ground state to the excited state and starts its nonadiabatic dynamics. The times t'_j are spread within the laser pulse intensity envelope. No ground-state adiabatic dynamics during the pulse are calculated – the ground-state density is considered to be stationary in PDA. We included a comment to clarify this point.

   > Page 10: The excitation time t'_j stands for the actual time within the laser pulse envelope when the trajectory is initiated in the excited electronic state with $\{\underline{\mathbf{R}}_j, \underline{\mathbf{P}}_j\}$ (in contrast, the vertical sudden approximation would initiate *all* trajectories at the excitation time $t' = 0$).

   > Page 10: We propose to start the simulations at time 0 and then shift them to t'_j to avoid repeated calculations of the same simulation just shifted to a different initial time. This way, the unique trajectories are used multiple times by shifting them to different t'_js. Note that the trajectory itself is considered fixed in the ground state until time t'_j, when it is promoted to the excited electronic state.

3. Regarding the numerical demonstrations: The authors plot in Fig 3 the first moment of the density in the excited state and its variance. To isolate error from the FSSH non-adiabatic method itself from the initialization procedure, should the density somehow effectively be normalized? I mean, the FSSH may predict a different transition probability to the ground-state than the exact quantum dynamics, effectively, and that would be fault of FSSH, not the fault of the authors' new initialization procedure. Likewise in Fig 4. A comment here would be useful.

   We agree with the Reviewer that FSSH+PDA and QD with explicit laser pulse transfer different amounts of density to the excited state. While we consider a full excited-state population with PDA, QD transfers only 0.05% of the density to the excited state in the system studied. Thus, the QD population plots in the SI were normalized to be compared to PDA. We have added this information to the SI. We note that Figures 3 and 4 mentioned by the Reviewer both depict only the expectation value of the excited-state density and its variance, which do not require a normalization (the normalization is inherently present in the formula for the expectation value):

   $$\langle R \rangle_{S_1} = \frac{\langle \psi_{S_1} | R | \psi_{S_1} \rangle}{\langle \psi_{S_1} | \psi_{S_1} \rangle} \tag{1}$$

   For the FSSH+PDA, we used the expression

   $$\langle R \rangle_{S_1} = \frac{\sum_{i=1}^{N} R_i}{N} \tag{2}$$

   Hence, no postprocessing/renormalization of the data was necessary and we compared quantities directly calculated from the dynamics in the main text. We included a paragraph in the SI to clarify this point.

   > Page S-21: While the comparison of $\langle R \rangle_{S_1}$ and $\langle \Delta R \rangle_{S_1}$ between QD and FSSH+PDA(W) can be done directly from the simulation data due to the presence of a normalization in the formula for an expectation value, the comparison of electronic populations shown

in this Supporting Information requires a normalization of the QD populations. As we mentioned at the beginning of this Section, the field intensity E_0 was set to trigger a maximum of 0.05% population transfer to the excited electronic state, ensuring that our simulations are in the weak-field limit. Contrarily, PDA considers only the promoted part of the density (0.05%) and ignores the part remaining in the ground state. Thus, the QD electronic state populations must be normalized such that they reflect only the excited part of the density. In practice, we have taken the maximum of the QD excited-state population (0.05%) and rescaled it to 1. Note that this rescaling strategy was only possible as the depopulation of the excited electronic state due to nonadiabatic transitions happens long after the pulse.

Report of Reviewer #2

It is a nice paper, well written and presenting interesting and timely development. Indeed, taking into account pulse shape is important problem. My only suggestion is to reference the paper "Floquet Hamiltonian for incorporating electronic excitation by a laser pulse into simulations of non-adiabatic dynamics" in Chemical Physics Volume 515, Pages 46-51, where another approach to taking into account excitation during the pulse shape has been suggested. In that paper it was demonstrated that excitation during the pulse can be significant and a method to take it into account has been suggested. The difference is that Floquet approach treats field quantum mechanically, not as a classical perturbation employed in this work. I believe the alternative Floquet technique would be worth mentioning here.
We thank the Reviewer for supporting the publication of our manuscript. We have not discussed approaches for explicit inclusion of the laser pulses in the Hamiltonian (where the Floquet theory belongs, although it is not included in the Hamiltonian in the form of electric field) as we focus on implicit approaches; therefore, omitting all references to that topic. We have included the reference mentioned by the Reviewer to our revised manuscript as well as references to other explicit methods when we mention them.

> Page 3: This observation can be rationalized by the associated computational cost of including a laser pulse explicitly in nonadiabatic dynamics[18–20] and ...

Report of Reviewer #3

The manuscript “Including photoexcitation explicitly in trajectory-based nonadiabatic dynamics at no cost” by Janoš, Slavíček and Curchod discusses the importance of including explicitly the effect of the photoexcitation in the initialization of a nonadiabatic process and develops a numerical strategy to do it in an efficient way for trajectory-based methods. The theory is developed succinctly but clearly in the main text and is supported by convincing numerical results describing the photodissociation of NaI and the photoisomerization of protonated formaldimine. In addition, the Supporting Information (SI) presents extensively the theoretical framework, includes computational details, and describes the algorithm. The major advance reported in this work is the strategy proposed by the authors. This strategy aims to include efficiently and accurately the effect of the excitation via a laser pulse in a trajectory-based simulation of excited-state dynamics. Such strategy is based on a rigorous quantum-mechanical description of the excitation processes treated within perturbation theory and allows the authors to derive an algorithm that is readily applicable. In addition, the importance of treating explicitly the excitation is demonstrated in two cases, namely for NaI and protonated formaldimine.

The immediate significance of this advance is the possibility of running simulations of nonadiabatic dynamics that provide and direct comparison with experiments of photochemistry, since they are able to describe quite realistically the experimental excitation process. In addition, the strategy proposed by the authors can be even used as a post-processing tool, for instance allowing to reuse existing trajectories without the need to rerun expensive nonadiabatic simulations.

My overall assessment of the manuscript is that it is an excellent work. Therefore, I can recommend publication in The Journal of Physical Chemistry Letters, provided that the authors address my comments below.
We thank the Reviewer for their positive evaluation of our work.

1. When it comes to the calculations of experimental signals, like the time-resolved electron

diffraction signal mentioned by the authors in the Introduction, I think that it is common practice to convolute the numerical results with a function accounting for the final duration of the pulse. Would the strategy developed in this work have a different effect on such a signal? More generally, can the authors clarify what the main differences can be expected to be between these two procedures to account for the effect of the pulse on observables?

This is correct and a convolution is often applied to the calculated observable in nonadiabatic molecular dynamics. The only issue is that the convolution function, and more specifically the width of the pulse, is not always defined in the same way. The PDA strategy allows us to rigorously define which FWHM should be used for the convolution and how to apply it to the calculated observable. This is what we did actually for the windowing and PDAW results presented in our manuscript too – the windowing selects a certain energy window and the convolution brings in the time spread of the laser pulse. As stated in the text, the PDAW approximates the PDA well for Gaussian pulses but starts to run out of steam for other types of pulses. We advocate for using the convolution defined in Eqs. (13) and (15) (main text) for the convolution. We note that an additional convolution may be required when the FWHM related to the probe pulse is larger than that of the pump – the PDA only focuses on the latter.

2a. The derivation of the promoted density is very elegant and convincing, at least as long as the quantum-mechanical case is discussed: the promoted density is a "modified" ground-state density, ie, modified by the squared of the interaction with the field and by the Wigner representation of the pulse.

Can the instantaneous-excitation limit be recovered from this expression?

The instantaneous (vertical) excitation limit can be recovered from the promoted density expression. Considering an instantaneous δ-pulse leads to the pulse Wigner transform equal to 1 at the excitation time t_0 and equal to zero at any other times. In other words, the full ground-state density (multiplied by the transition dipole moment) will be promoted instantaneously at one time t_0. We mention the instantaneous excitation in terms of promoted density in footnote *d* on page 10.

2b. To translate the concept of promoted density from the quantum-mechanical formulation to the classical formulation is terms of trajectories, it seems to me that some sort of reweighting procedure is developed. Specifically, the ground-state initial conditions sampled without the field are promoted according to some quantity/probability, namely the squared of the interaction with the field and by the Wigner representation of the pulse. Les me consider for the moment only the simple case of a non-negative Wigner representation. Is this quantity, called p in the manuscript, a probability? Is it defined between 0 and 1? Also, the promoted probability density depends on the nuclear positions and on time (the time at which the excitation of a particular trajectory takes place): is this actually a probability density in the "extended" configuration-time space? While the actual numerical procedure introduced by the authors, that randomly select an initial condition and an initial time to perform the photoexcitation, seems effective, I have difficulties in justify it in light of the quantum-mechanical equations.

We thank the Reviewer for this good point. The promoted density (we note we have not called it promoted probability density) is not a probability density; it would not integrate to 1. Hence, the quantity called p is also strictly speaking not a probability. Both quantities correspond to the transition probability and depend on the field strength E_0. The promoted density would, therefore, integrate to a value smaller than 1 and be proportional to the population transfer from ground to the excited state. For our QD simulations with explicit field, the population transfer was always below 0.05% (the 99.95% of the ground-state density remained unperturbed during the dynamics). Contrarily, the PDA was devised such that it considers only the promoted part of the density (0.05%) and ignores the part remaining in the ground state. This is connected to the quantity p which is proportional to the excitation probability. By 'renormalizing' it with a quantity $p_{\max}$ (maximum population transfer probability), we make p effectively a probability in PDA. We note that the QD electronic state populations must be normalized in a similar manner so that they reflect only the excited part of the density. We have added a short discussion in the SI on this issue, also inspired by the question 3 of Reviewer 1 (see answer above), and also several modifications to the main text.

> Page 5: The promoted nuclear density is the central quantity of our derivation and represents the nuclear density promoted to the excited electronic state at time t', which we call the excitation time. As such, ρ_p is proportional to the interaction strength represented as E_0.

2c. When moving from the full quantum treatment to the semiclassical treatment that introduces

the phase-space representation in Eq.(7), do the authors actually do the Wigner transform of Eq.(3) or do they replace every operator/function in Eq.(3) with its phase-space representation? I guess that the former would yield a more complicated expression than Eq.(7), and in any case this should be clarified in the text.
We apply the Wigner transform but truncated to the lowest order of $\hbar$; hence, obtaining such simple expression. The full Wigner transform of the density operator would be, indeed, much more complicated expression. This strategy is equivalent to just replacing the quantum operators by their classical counterparts and follows the procedure performed by Engel and Meier in their work on the promoted density. We modified the part in the main text accordingly.

> Page 5/6: Finally, inserting the promoted density into the expression for the excited-state nuclear density (Eq. (3)) and taking a classical limit by retaining only the lowest-order term in $\hbar$ in the Wigner transform of the density operator results in the final formula for PDA, ...

3. The promoted density approach (PDA) is applicable efficiently as a post-processing tool because it is based on the assumption that the photoexcitation process and the dynamics in the excited state can be disentangled. However, I would expect that some trajectories are transferred back to the ground state and perhaps have an effect on the overall nonadiabatic dynamics. Can this effect appear in the formalism developed in the manuscript?
The Reviewer is correct that trajectories can transfer back to the ground state after photoexcitation during the following nonadiabatic dynamics. From this perspective, PDA is derived to model only the part of the population transfer from the ground-state density induced by the laser pulse. The following nonadiabatic dynamics are hidden in the Liouvillian propagator acting on the promoted density, being fully decoupled from the excitation process. This is one of the limitations of our approach, yet the developed scheme was not intended to tackle complex situations like this, but rather provide a simple "first-order" scheme for including laser pulses.

4. The PDA is reminiscent of the approach developed by Barbatti in 10.1021/acs.jctc.0c00501, even if that was developed to account for a photoexcitation by incoherent light rather than a laser pulse. Can the authors comment on the formal and algorithmic analogies/differences of the two approaches?
The work by Barbatti presents an interesting approach for continuous thermal radiation and bears some analogies, yet we do not find it profoundly similar to PDA. Let us briefly summarize Barbatti's approach named mixed quantum-classical dynamics with pulse envelopes (MQC-PE). MQC-PE is based on the Chenu–Brumer formula for thermal radiation which represents the continuous thermal radiation by an incoherent sum of short laser pulses coming one after the other. For the case of sunlight, these laser pulses are about 5 fs long having a broadband spectrum. The ultrashort pulses are then treated individually with the vertical excitation approach; citing the original article 'Moreover, the ultrashort pulse of each realization at high temperatures plays favourably for the validity of the instantaneous approximation.'. Thus, there is no temporal effect of the individual pulses involved. The initial conditions for vertical excitation are weighted with the excitation probability calculated using the spectrum of the radiation and the transition dipole moment. This weighting bears a similarity with the windowing defined in PDAW, yet that is the only similarity we could find. Since the thermal radiation is described by a series of consequent laser pulses, the instantaneous excitation mentioned above is repeated for each laser pulse in time, which creates a constant flux of trajectories to the excited state. There are not many similarities in both the theory and the algorithm. PDA aims to tackle the effect of ultrashort laser pulses trying to resolve the dynamics on the shortest timescales. The main goal is to describe fine effects of laser pulses, such as their duration, frequency or chirp, on the subsequent nonadiabatic dynamics. On the other hand, MQC-PE targets long time dynamics triggered by continuous thermal radiation which does not change (the only parameter is the temperature of the source). Inherently, MQC-PE uses instantaneous vertical approximation which PDA tries to alleviate. Hence, we do not mention the work in our manuscript as it is not directly related to excitation by short coherent laser pulses.

5. Technical and minor questions:

   In Eq.(1) and related expressions (also in the SI), Vint appears as an operator on the left-hand side but the symbol of operator $\wedge$ disappears on the right-hand side, I guess because it is only multiplicative in nuclear space. Perhaps this choice of convention should be defined somewhere in the text.

We have emphasized in the manuscript that we derive PDA in the position representation and, therefore, electronic energies and transition dipole moments are only multiplicative factors.

> Page 4: We consider a molecular system with a ground (g) and an excited (e) electronic state, coupled through a weak interaction defined in the position representation as ...

> Page S-9: Note that the derivation is performed in the position representation; therefore, operators depending only on $\underline{\mathbf{R}}$ do not bear the $\wedge$ in our notation.

6. At the end of page 4, the text says that ρ_g is stationary: under which Hamiltonian? I guess $\hat{H}_g$.
The Reviewer is correct, ρ_g is stationary under the $\hat{H}_g$ Hamiltonian. We have modified the sentence in the main text accordingly.

> Page 4: The ground-state nuclear density, ρ_g, is considered to be stationary under the $\hat{H}_g$ Hamiltonian.

7. I am not convinced that Eq.(28) in the SI is simply the nuclear wavefunction, because it is written in the electronic basis, so in my opinion this is a representation of the molecular state.
Eq. (28) defines the nuclear amplitudes (wavefunctions) on the ground and excited electronic states and as such it represents a molecular state. However, we do not call it a molecular wavefunction as it does not directly include the electronic wavefunctions (in a Born-Huang sense).

8. In the derivation described in Section 3 of the SI, I think that sometimes the authors use 0 as initial time and sometimes t0 (see for instance Eq.(34)).
We thank the Reviewer for pointing out the inconsistency in Eq. (34) and the remaining of the text. We use both time t_0 and time 0 ($t = 0$) in our derivation, yet we did so inconsistently. The time t_0 is the initial time for the perturbation theory formula, while time 0 is a time where the time-zero density ρ_z is defined. We have corrected the text and added an explanatory comment about the times t_0 and 0.

> Page S-9: [a]To clarify, the propagation does not go from t' back to time t_0 but to the time zero ($t = 0$). Nevertheless, we could backpropagate to an arbitrary time given we would then propagate from this arbitrary time back to the time t. In the end, the operator acting is $\mathrm{e}^{-\frac{i}{\hbar}\hat{H}_e(t-t')}$.

9. To derive Eq.(49) in the SI, the first step is to exponentiate the logarithm of the product of the transition dipole moment and of the field, which a function with many divergences, ie, every time the cosine is zero. Can the authors propose another derivation that does not imply introducing divergences in an initially well-behaving expression?
We agree with the Reviewer that exponentiating the logarithm might be mathematically problematic if the argument of the logarithm approaches zero. However, the function within the logarithm is not time-dependent (it does not bear a cosine function) but it is merely the scalar product of the transition dipole moment and $\vec{E}_0 = E_0\vec{\lambda}$, where E_0 is the electric field magnitude and $\vec{\lambda}$ stands for the field polarization. Thus, this function is time-independent and equal to zero only if the transition dipole moment is zero or the projection onto the field polarization is zero. In both cases, there would be no population transfer. While we agree that this mathematical step is not ideal, we argue that the argument equals zero only in cases when the interaction term is zero and there is no population transfer by definition.

10. Typo: In the SI just before Eq.(26), I think that “vector field” should be replaced by “vector potential”.
We thank the Reviewer for noticing the typo, we have corrected it in the SI.

> Page S-7: Returning to pulses defined by a pulse envelope, one can consider a vector potential in the following form ...

We thank again the Reviewer for their thorough assessment of our work. Their comments also stimulated us to improve the description of how PDA describes the excited-state density during the pulse. As a result, we modified the main text as follows (and adapted the SI accordingly):

> Page 6: Eq. (7) as such is derived to describe the excited-state nuclear density following the interaction with the laser pulse (see SI for a detailed discussion). However, we can extend the reach of Eq. (7) such that it describes the excited-state density also during the pulse by altering the upper integration limit to t instead of ∞,

$$\rho_e^{\mathrm{cl}}(\underline{\mathbf{R}}, \underline{\mathbf{P}}, t) = \frac{1}{\hbar^2}\int_{-\infty}^{t} \mathrm{e}^{\mathcal{L}_e^{\mathrm{cl}}(t-t')} \left[|\vec{\mu}_{eg}(\underline{\mathbf{R}}) \cdot \vec{E}_0|^2 \mathcal{W}_E(t', \Delta E_{eg}^{\mathrm{el}}(\underline{\mathbf{R}})/\hbar)\rho_g^{\mathrm{cl}}(\underline{\mathbf{R}}, \underline{\mathbf{P}})\right] \mathrm{d}t' \, . \quad (8)$$

This modification means that only the contributions of the promoted density to ρ_e coming from times before the current time t are included. Any contributions to ρ_e coming from the promoted density at later times $t' > t$ are neglected. This empirical alteration of the upper integration limit affects the overall equation only during the pulse interaction and not for times t after the pulse. This modification allows PDA to describe the excited-state nuclear density *within* the pulse duration while not altering the final excited-state density after the pulse. More details on this procedure (and its numerical validation) are provided in the SI.

Manuscript Formatting Request - Non-scientific changes

---

1. Incomplete references:
   -Please include at least the first page number for the following incomplete journal reference: 18.
   -Please include date of access for the following incomplete Website reference: 31.
   We have modified the references accordingly.
2. The TOC graphic should fit in an area no larger than 3.25 in. × 1.75 in. (approx. 8.25 cm × 4.45 cm) and should have adequate resolution and clarity. Confirm that all text is legible at this size.
   We confirm that the TOC graphic fits in the designated area and the text is legible at this size.

We note that we modified the description of the PDA code as we have turned it into a standalone Python package available to the community through Python package installers such as pip.

We hope that the revised version of our manuscript is now suitable for publication in The Journal of Physical Chemistry Letters.

Yours Sincerely,

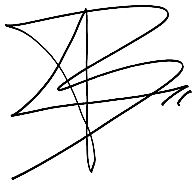

Basile F. E. Curchod
